# Microbial Transformation and Biological Activities of the Prenylated Aromatic Compounds from *Broussonetia kazinoki*

**DOI:** 10.3390/molecules27061879

**Published:** 2022-03-14

**Authors:** EunA Choi, Fubo Han, Jisu Park, Ik-Soo Lee

**Affiliations:** College of Pharmacy, Chonnam National University, Gwangju 61186, Korea; 206412@jnu.ac.kr (E.C.); hanfubo0306@gmail.com (F.H.); qkrwltn9410@naver.com (J.P.)

**Keywords:** *Broussonetia kazinoki*, microbial transformation, tyrosinase inhibition, cytotoxicity

## Abstract

*Broussonetia kazinoki* has been used as a traditional medicine for the treatment of burns and acne, and its extracts have been found to show tyrosinase inhibitory and anticancer activities. In this study, the tyrosinase inhibitory and cytotoxic activities of *B. kazinoki* were explored, leading to the isolation of kazinol C (**1**), kazinol E (**2**), kazinol F (**3**), broussonol N (**4**), and kazinol X (**5**), of which the compounds **4** and **5** have not been previously reported. Microbial transformation has been recognized as an efficient tool to generate more active metabolites. Microbial transformation of the major compounds **1** and **3** was conducted with *Mucor hiemalis*, where four glucosylated metabolites (**6**–**9**) were produced from **1**, while one hydroxylated (**10**) and one glucosylated (**11**) metabolites were obtained from **3**. Structures of the isolated metabolites were determined by extensive spectroscopic analyses. All compounds were evaluated for their tyrosinase inhibitory and cytotoxic activities. Compound **3** and its metabolites, kazinol Y (**10**) and kazinol F-4″-*O*-β-d-glucopyranoside (**11**), exhibited the most potent tyrosinase inhibitory activities with the IC_50_ values ranging from 0.71 to 3.36 µM. Meanwhile, none of the metabolites, except for kazinol C-2′,3″-di-*O*-β-d-glucopyranoside (**7**), showed moderate cytotoxic activities (IC_50_ 17.80 to 24.22 µM) against A375P, B16F10 and B16F1 cell lines.

## 1. Introduction

*Broussonetia kazinoki*, a deciduous shrub tree belonging to the family Moraceae, is widely distributed throughout Korea, China, and Japan [1,2,3]. Since ancient times, its leaves, branches, roots and fruits have been used for various therapeutic purposes including the amelioration of vision, and suppression of edema [1,2,3,4]. In addition, it has been traditionally used for dermatologic diseases such as burns and acne in Korea according to the *Principles and Practice of Eastern Medicine*, an encyclopedia of medical knowledge [5,6]. Previous biological investigations have demonstrated that *B. kazinoki* exhibited a variety of pharmacological effects, such as antioxidant, anti-inflammatory, anticancer, anti-allergic, anti-diabetic, and anti-hyperglycemic activities [2,3,4,5,6,7]. Moreover, the extract of *B. kazinoki* has been registered as a skin-whitening agent by the Korea Food and Drug Administration (KFDA) due to its potent tyrosinase inhibitory and anti-melanogenic effects [5,8]. Additionally, a series of prenylated polyphenols including kazinol F and broussonin C isolated from *B. kazinoki* have been reported to exhibit significant tyrosinase inhibitory effects [8].

Tyrosinase (EC 1.14.18.1), also known as catecholase or diphenol oxidase, is a multifunctional copper-containing enzyme and is widely distributed in plants, fungi, bacteria and animals [9,10]. It catalyzes the hydroxylation of L-tyrosine to L-DOPA (3,4-dihydroxy-L-phenylalanine) and the subsequent oxidation of L-DOPA to L-dopaquinone [10]. Then, the dopaquinone forms melanin through polymerization with a series of enzymatic and nonenzymatic reactions [11,12]. Melanin is responsible for skin color and plays an important role in human skin, for example, the prevention of skin injury under normal physiological conditions [12]; however, an overproduction and accumulation of melanin can result in hyperpigmentary disorders of the skin, such as freckles, melasma, age spots, and melanoma [9]. More seriously, tyrosinase catalyzes the formation of neuromelanin, which is associated with neurodegenerative disorders like Parkinson’s disease [10,11,13]. Therefore, inhibiting tyrosinase activity applies to the treatment of pigmentation disorders associated with melanin hyperpigmentation and some related diseases [9,14]. Though a number of well-known tyrosinase inhibitors, like arbutin, kojic acid, and hydroquinone, have been reported from natural or synthetic sources during the past few decades, their applications have been limited due to serious side effects such as dermatitis, cytotoxicity and hepatotoxicity [9,14]. Thus, it is desirable to find new tyrosinase inhibitors with improved safety.

Microbial transformation is known as a useful method to generate more active derivatives with minor structural modifications in bioactive substrates using the metabolic activities of microorganisms [15,16]. The transformation is accomplished by a series of enzymatic reactions including hydroxylation, oxidation, and glycosylation under mild conditions [16,17,18,19]. Furthermore, microbial transformation is considered to be an environmentally friendly tool and has been used successfully to produce pharmaceuticals from natural products [15,16].

**Figure 1:**
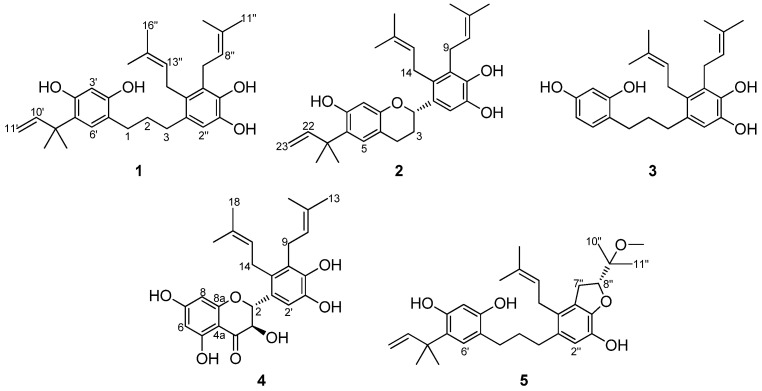
Chemical structures of compounds **1**–**5**.

In the present study, we isolated and characterized the constituents of the root barks of *B.* *kazinoki* with tyrosinase inhibitory properties based on the bioactivity-guided fractionation process (Figure 1). Further transformation of kazinols C (**1**) and F (**3**) was performed with *Mucor hiemalis*, which led to the isolation of five glucosylated and one oxidized metabolite (**6**–**11**). All of the isolated metabolites were evaluated for their tyrosinase inhibitory and cytotoxic activities, and it was revealed that kazinol F-4″-*O*-β-d-glucopyranoside (**11**) had the strongest anti-tyrosinase activity with no cytotoxic activity against melanoma cells.

## 2. Results and Discussion

### 2.1. Structure Elucidation of Compounds from Broussonetia kazinoki

To isolate and identify the secondary metabolites of *B. kazinoki* for anti-tyrosinase activity, the EtOH extract of its root barks was investigated [8]. The CH_2_Cl_2_ fraction of the EtOH extract showed promising tyrosinase inhibitory effects, and the subsequent activity-guided fractionation led to the isolation of five prenylated polyphenols **1**–**5** (Figure 1). Structures of the compounds were determined using the 1D- and 2D-NMR (nuclear magnetic resonance), including COSY (correlation spectroscopy), HSQC (heteronuclear single quantum correlation), and HMBC (heteronuclear multiple bond correlation), as well as mass spectroscopic data analyses. The three known compounds were identified and confirmed as kazinol C (**1**), kazinol E (**2**), and kazinol F (**3**) by comparison of their spectroscopic data with those reported in the literatures [8,20]. 

**Figure 2:**
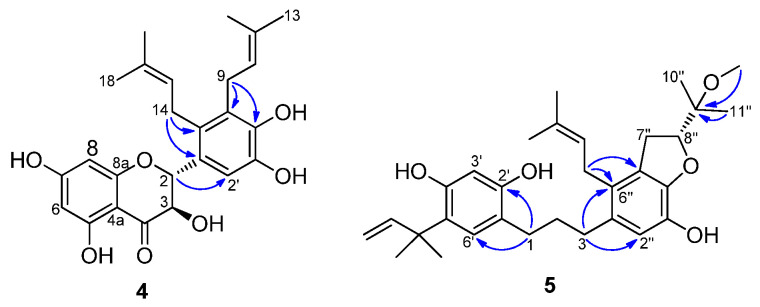
Key HMBC correlations (^1^H→^13^C) of compounds **4** and **5**.

Compound **4** showed a molecular formula of C_25_H_28_O_7_ by the HRFDMS peak at *m*/*z* 440.1841 [M]^+^ (calcd. 440.1835). The UV spectrum of **4** exhibited two distinct peaks at 210 and 291 nm, characteristic of a flavanonol system. The ^1^H NMR spectrum of compound **4** displayed: three aromatic proton signals at δ_H_ 7.05 (1H, s), 5.95 (1H, s), and 5.89 (1H, s); two sets of prenyl group proton signals at δ_H_ 5.12 (1H, t, *J* = 6.6 Hz), 5.08 (1H, t, *J* = 6.1 Hz), 3.46 (1H, dd, *J* = 6.1, 16.4 Hz), 3.40 (2H, d, *J* = 6.2 Hz), 3.33 (1H, dd, *J* = 6.1, 16.4 Hz), 1.75 (3H, s), 1.66 (3H, s), 1.62 (3H, s), and 1.61 (3H, s); and a set of AB-type proton signals at δ_H_ 5.31 (1H, d, *J* = 11.5 Hz), and 4.64 (1H, t, *J* = 11.5 Hz) due to H-2 and H-3. The positions of the two prenyl groups were determined on the basis of its HMBC spectrum (Figure 2). The long-range correlations from H-9 to C-4′/5′, H-14 to C-1′/6′, and H-2 to C-2′ indicated that two prenyl groups were adjacent to each other and attached at the C-5′ and C-6′ positions of ring B. The large coupling constant (11.5 Hz) between H-2 and H-3 indicated that ring C had a 2,3-*trans*-configuration, not a *cis*-configuration which gave a small *J* value (~2 Hz) [3], and the absolute configuration of compound **4** was considered to be 2*R*,3*R* based on its positive specific rotation (${\left[\alpha \right]}_{D}^{20}\;$+74.6°) by comparison with the reported data of related structures [3,21]. Based on the above analysis, the structure of compound **4** was assigned (2*R*,3*R*)-3,5,7,3′,4′-pentahydroxy-5′,6′-diprenylflavanonol, and was named broussonol N.

Compound **5** exhibited a molecular formula of C_31_H_42_O_5_ by its HRFDMS peak at *m*/*z* 494.3034 [M]^+^ (calcd. 494.3032). The UV spectrum of **5** exhibited absorption maxima at 228 and 285 nm, which was similar to the kazinol, indicating that it was also a 1,3-diphenylpropane. The ^1^H NMR spectrum of compound **5** revealed: three aromatic proton signals at δ_H_ 6.93 (1H, s), 6.61 (1H, s), and 6.32 (1H, s); a 1,1-dimethylallyl group proton signal at δ_H_ 6.17 (1H, dd, *J* = 10.6, 17.7 Hz), 5.32 (1H, dd, *J* = 0.6, 17.7 Hz), 5.26 (1H, dd, *J* = 0.6, 10.6 Hz), and 1.39 (6H, s); one prenyl group proton signal at δ_H_ 5.01 (1H, t, *J* = 6.6 Hz), 3.16 (2H, d, *J* = 6.6 Hz), 1.70 (3H, s), and 1.67 (3H, s); and the signals of a 1,3-disubstituted propane moiety at δ_H_ 2.59 (2H, m), 2.54 (2H, m), and 1.81 (2H, m). In addition, the proton signals of a 2-(1-methoxy-1-methylethyl)-dihydrofuran moiety at δ_H_ 4.73 (1H, t, *J* = 9.2 Hz), 3.31 (3H, s), 3.09 (2H, dd, *J* = 9.6, 15.8 Hz), 1.24 (3H, s), and 1.20 (3H, s) were also observed in **5**, which was quite similar to that of kazinol T [8], except for the presence of a methoxy group instead of a hydroxyl group at the C-9″ position. This was supported by the long-range correlation from 9″-OCH_3_ to C-9″ (δ_C_ 77.03) (Figure 2). The absolute configuration of compound **5** at C-9″ was considered to be *R* based on its negative specific rotation (${\left[\alpha \right]}_{D}^{20}$ −6.31°) by comparison with the reported data of related structures [18,22,23]. Based on the above analysis, the structure of compound **5** was assigned to be 5′-(2-methylbut-3-en-2-yl)-6″-(3-methylbut-2-enyl)-4″,5″-[(*R*)-2-(1-methoxy-1-methylethyl)]-dihydrofuranyl-2′,4′,3″-trihydroxydiphenylpropane, and was named kazinol X.

### 2.2. Microbial Transformation of Kazinols C and F by Mucor hiemalis

A total of 14 microbial cultures were screened to evaluate their ability to metabolize the isolated compounds **1** and **3** under a standard two-stage fermentation procedure [18,19]. Based on the analysis of TLC plates involving the substrates and culture controls, it was observed that *Gliocladium deliquescens* and *Mucor hiemalis* showed the ability to metabolize **1**, and *Alternaria alternata,* *Absidia coerulea*, *G. deliquescens* and *M*. *hiemalis* showed the ability to metabolize **3** (Supplementary Table S1). Among the active strains, the fungus *M. hiemalis* was selected for preparative-scale fermentation studies since it exhibited the highest transformational capability towards **1** and **3**. The subsequent transformation studies led to the production of five glucosylated and one oxidized metabolite (**6**–**11**). 

**Figure 3:**
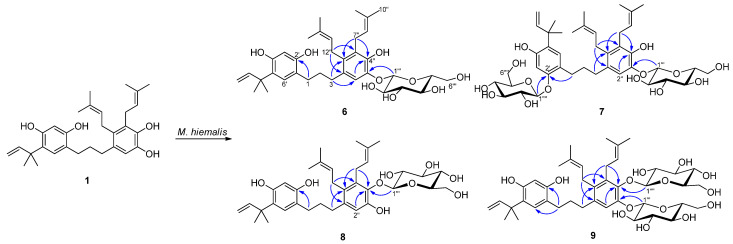
Metabolites of kazinol C (**1**) transformed by *M. hiemalis*. Selected HMBC correlations (^1^H→^13^C) are indicated by arrows.

Compound **6** had a molecular formula of C_36_H_50_O_9_, as established by its HRFDMS peak at *m*/*z* 626.3439 [M]^+^ (calcd. 626.3455), which was one glucose unit higher than that of **1**, indicating that **6** was a glucosylated derivative of **1**. This was supported by the occurrence of six new carbon signals in the ^13^C NMR spectrum of **6**, including five methine carbon resonances at δ_C_ 103.4, 77.1, 75.8, 73.4, 69.7, and one methylene carbon at δ_C_ 60.7. The corresponding proton signals at δ_H_ 4.50 (1H) and 3.69–3.19 (6H) were observed in the ^1^H NMR spectrum. All of these data were consistent with previous reports on the d-glucose moiety [19,24,25], and the glucose was determined to be in a β-configuration by the large coupling constant (7.1 Hz) of the anomeric proton signal at δ_H_ 4.50 (H-1‴). The significantly downfield-shifted aromatic proton signal at δ_H_ 6.81 (H-2″) suggested the glucose moiety was attached to C-3″ through an ether linkage. This was confirmed by a long-range correlation between the anomeric proton signal at δ_H_ 4.50 (H-1‴) and carbon signal at δ_C_ 143.1 (C-3″) in the HMBC spectrum (Figure 3). Thus, compound **6** was assigned to be kazinol C-3″-*O*-β-d-glucopyranoside.

Compound **7** had a molecular formula of C_42_H_60_O_14_, as established by the peak at *m*/*z* 788.3997 [M]^+^ (calcd. 788.3983) in its HRFDMS spectrum, which was one glucose unit higher than that of **6**, indicating that two glucose units had been introduced into the molecule of **1**. The additional proton signals at δ_H_ 4.64, 4.51 and 3.71–3.18 (12H), as well as the carbon signals at δ_C_ 103.5, 101.5, 77.1, 76.9, 76.8, 75.8, 73.5, 73.4, 69.7, 69.6, 60.7, and 60.7 observed in the ^1^H and ^13^C NMR spectra of **7**, indicated the presence of two D-glucose residues [24,25]. The glucose units were identified as β-glucose by their large coupling constants (6.5 Hz and 6.6 Hz) of the anomeric proton signals δ_H_ 4.64 and 4.51, respectively. The significantly downfield-shifted aromatic proton signals at δ_H_ 6.60 (H-3′) and 6.82 (H-2″) suggested the two glucose moieties were linked to C-2′ and C-3″, respectively. These connections were confirmed by the long-range correlations between the anomeric proton signal at δ_H_ 4.51 (H-1‴) and the carbon signal at δ_C_ 142.9 (C-3″), as well as between δ_H_ 4.64 (H-1″″) and δ_C_ 154.1 (C-2′) in the HMBC spectrum of **7** (Figure 3). Thus, compound **7** was assigned to be kazinol C-2′,3″-di-*O*-β-d-glucopyranoside.

Compound **8** had a molecular formula of C_36_H_50_O_9_, as deduced from the peak at *m*/*z* 626.3469 [M]^+^ (calcd. 626.3455) in its HRFDMS spectrum, which was one glucose unit higher than that of **1**, indicating that **8** was also a monoglucosylated derivative of **1**. The additional proton signals at δ_H_ 4.42 and 3.64–3.15 (6H), as well as the carbon signals at δ_C_ 105.9, 77.3, 76.2, 74.1, 69.5, and 60.8 in the ^1^H and ^13^C NMR spectra of **8**, indicated the presence of a D-glucose residue. The coupling constant (7.6 Hz) of anomeric proton (δ_H_ 4.42) in the ^1^H NMR spectrum of **8** indicated a β-configuration of this glucose moiety. The significantly downfield-shifted aromatic carbon signals at δ_C_ 147.0 (C-3″) and 133.9 (C-5″) suggested this glucose moiety was attached to C-4″, and it was confirmed by the correlation between H-1‴ and C-4″ in the HMBC spectrum of **8** (Figure 3). Thus, compound **8** was assigned to be kazinol C-4″-*O*-β-d-glucopyranoside.

Compound **9** had a molecular formula of C_42_H_60_O_14_, as established by the peak at *m*/*z* 788.4016 [M]^+^ (calcd. 788.3983) in its HRFDMS spectrum, which was one glucose unit higher than that of **8**, indicating that two glucose units had been introduced into the molecule of **1**. The additional proton signals at δ_H_ 4.85, 4.65 and 3.69–3.02 (12H), as well as the carbon signals at δ_C_ 103.5, 102.3, 77.1, 77.1, 76.5, 75.8, 74.3, 73.5, 70.0, 69.8, 61.1, and 60.7 in the ^1^H and ^13^C NMR spectra of **9**, indicated the presence of two D-glucose residues. The coupling constants (7.4 Hz and 7.6 Hz) of the anomeric protons (δ_H_ 4.85 and 4.64) in the ^1^H NMR spectrum of **9** indicated the β-configuration of these two glucose moieties. The aromatic proton signals of ring A at δ_H_ 6.70 (s, H-6′) and 6.29 (s, H-3′) were quite similar to those of compound **1**, and together with the significantly downfield-shifted aromatic proton signal at δ_H_ 6.93 (H-2″) suggested that these two glucose moieties should be linked to C-3″ and C-4″. These connections were confirmed by the cross-peaks of H-1‴ and C-3″ as well as H-1″″ and C-4″ in the HMBC spectrum of 10 (Figure 3). Based on the above analyses, compound **9** was assigned to be kazinol C-3″,4″-di-*O*-β-d-glucopyranoside.

**Figure 4:**
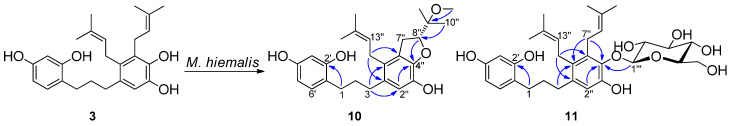
Metabolites of kazinol F (**3**) transformed by *M. hiemalis*. Selected HMBC correlations (^1^H→^13^C) are indicated by arrows.

Compound **10** showed a [M]^+^ peak at *m*/*z* 426.2414 (calcd. for C_26_H_34_O_5_, 426.2406) in its HRFDMS spectrum, which established a molecular formula of C_26_H_34_O_5_. Significant differences were observed in the ^1^H and ^13^C NMR spectra of **10** compared with those of **3**. The highly downfield-shifted oxymethine proton signal at δ_H_ 4.58 (1H, t, *J* = 9.1 Hz) together with the corresponding carbon signal at δ_C_ 89.3 indicated that a dihydrobenzofuran group was formed from the prenyl group substituted on ring B [26]. The spectroscopic data of **10** were quite similar to those of kazinol V [22], except for the signal of the methoxy group, which was supposed to be located at the C-9″ position. The location of the additional methoxy group was confirmed by the cross-peak of the proton signal at δ_H_ 3.16 (3H, s) and carbon signal at δ_C_ 77.5 in its HMBC spectrum (Figure 4). The absolute configuration of compound **10** at C-8″ was considered to be *R* based on its negative specific rotation (${\left[\alpha \right]}_{D}^{20}$ −12.38°) by comparison with the reported data of related compounds which had a dihydrofuran group in their structures [18,22,23]. Based on the above analyses, compound **10** was assigned to be 6″-(3-methylbut-2-enyl)-4″,5″-[(*R*)-2-(1-methoxy-1-methylethyl)]-dihydrofuranyl-2′,4′,3″-trihydroxydiphenylpropane, and was named kazinol Y. 

Compound **11** had a molecular formula of C_31_H_42_O_9_, as deduced from the peak at *m*/*z* 558.2839 [M]^+^ (calcd. 558.2829) in its HRFDMS spectrum, which was one glucose unit higher than that of **3**, indicating that **11** was also a monoglucosylated derivative of **3**. The additional proton signals at δ_H_ 4.43 and 3.65–3.16 (6H), as well as the carbon signals at δ_C_ 105.9, 77.3, 76.2, 74.1, 69.6, and 60.8 in the ^1^H and ^13^C NMR spectra of **11**, indicated the presence of a D-glucose residue. The coupling constant (7.7 Hz) of the anomeric proton (δ_H_ 4.43) in the ^1^H NMR spectrum of **11** indicated a β-configuration of this glucose moiety. The significantly downfield-shifted aromatic carbon signals at δ_C_ 147.1 (C-3″) and 133.9 (C-5″) suggested this glucose moiety was attached to C-4″, and it was confirmed by the correlation between H-1‴ and C-4″ in the HMBC spectrum of **11** (Figure 3). Thus, compound **11** was assigned to be kazinol C-4″-*O*-β-d-glucopyranoside.

### 2.3. Tyrosinase Inhibitory Activity

All the compounds were investigated for their tyrosinase inhibitory effects using L-tyrosine as the substrate. Kojic acid, a well-known tyrosinase inhibitor currently used in cosmetics as a skin-whitening agent, was used as a positive control. Metabolite **11** exhibited the most potent inhibitory effect against tyrosinase (IC_50_, 0.71 μM), followed by its parent compound **3** and metabolite **10**, with IC_50_ values of 2.12 and 3.36 μM, respectively (Table 1). Compound **4**, which also exhibited a stronger inhibitory effect than the kojic acid, showed moderate activity with an IC_50_ value of 24.11 μM. All of the other compounds, which have a 1,1-dimethylallyl group in their A-ring, were considered to be inactive, as their IC_50_ values were over 80 μM. Moreover, it has been reported that kazinol F and broussonin C have exhibited much stronger tyrosinase inhibition than the ring A-prenylated 1,3-diphenylpropanes [8], and similar results were observed in studies reporting the flavonoids as tyrosinase inhibitors from *Broussonetia papyrifera* [27]. The above analyses indicated that prenylation in the ring A of the compounds with a 1,3-diphenylpropane skeleton might weaken their tyrosinase inhibition activities.

### 2.4. Cytotoxic Activity

To evaluate the anticancer potential of compounds **1**–**11**, an MTT assay was used to determine the cell viability of human melanoma (A375P) and murine melanoma (B16F10 and B16F1) cell lines following a 24 h treatment with compounds **1**–**11**. From the results shown in Table 1, it was observed that compounds **1**–**4** and metabolite **7** displayed potent cytotoxic effects against all cancer cell lines tested. Compounds **5** and **10** exhibited quite weak cytotoxicity compared to compounds **1** and **3**, suggesting that cyclization between the prenyl moiety and the adjacent phenolic hydroxyl group might decrease the cytotoxic activity. This was similar to the result that kazinol R, possessing a pyran ring in its structure, exhibited weaker cytotoxic effects than kazinol Q [28]. In addition, metabolite **7**, which had a glucose moiety in the ring A of its structure, showed much stronger activities than the other glucosylated metabolites, suggesting that *O*-glycosylation of 1,3-diphenylpropanes in the ring A might enhance their cytotoxic activities.

## 3. Materials and Methods

### 3.1. General Experimental Procedures

The NMR spectra were recorded in CDCl_3_, acetone-*d*_6_ or DMSO-*d*_6_ on Varian Unity Inova 500 and 600 spectrometers (Varian, Palo Alto, CA, USA) and a Bruker Avance Ⅲ HD 400 spectrometer (Bruker, Billerica, MA, USA), using TMS as the internal standard. The chemical shift values (δ) are reported in ppm units, and the coupling constants (*J*) are in Hertz (Hz). Optical rotations and IR spectra were measured with a Perkin Elmer 343 Plus polarimeter and a Perkin Elmer Spectrum 400 FT-IR/FT-NIR spectrometer (Waltham, MA, USA), respectively. HRFDMS was performed on a JEOL GC-MS: JMS-T200GC AccuTOF GCx-plus High Performance Gas Chromatograph—Time-of-Flight Mass Spectrometer (Seoul, Korea). TLC analyses were carried out on precoated silica gel 60 F_254_ glass plates (Merck, Darmstadt, Germany). Visualization of the TLC plates was performed under UV light (254 and 365 nm) and using an anisaldehyde-H_2_SO_4_ spray reagent followed by heating (120 °C, 1 min). The adsorbents used for the open column chromatography were Intertec silica gel 70–230 mesh (Intertechnologies Co., Ltd., Seoul, Korea) and Sephadex LH-20 (Amersham *Pharmacia Biotech* AB, Uppsala, Sweden). The HPLC was performed on a Waters 515 HPLC pump connected to a Waters 996 Photodiode Array detector (Waters Corp., Milford, MA, USA) using a Phenomenex Luna C_18_ column (25 cm × 10 mm) with HPLC grade methanol and water.

### 3.2. Materials and Microorganisms

The root barks of *Broussonetia kazinoki* were collected and identified by Eden farm in Jeonju, Korea, in August 2020, and a voucher specimen has been deposited at the College of Pharmacy, Chonnam National University. The microorganisms were obtained from the Korean Culture Center of Microorganisms (KCCM) and Korean Collection for Type Cultures (KCTC). Fourteen cultures were used for the preliminary screening procedure and are listed below: *Aspergillus oryzae* KCCM 60345, *Absidia coerulea* KCTC 6936, *Alternaria alternata* 6005, *Aspergillus fumigatus* 6145, *Cunninghamella elegans* var. *elegans* 6992, *Gliocladium deliquescens* 6173, *Glomerella cingulata* 6075, *Hormoconis resinae* 6966, *Monascus rubber* 6122, *Mortierella ramanniana* var. *angulispora* 6137, *Mucor hiemalis* 26779, *Fusarium merismoides* 6153, *Penicillium chrysogenum* 6933, and *Trichoderma koningii* 6042. The cultures of the microorganisms were stored at −60 °C with 20% glycerol.

Two types of media were used in the screening experiments and are listed below: *A. coerulea, A. alternata, A. fumigatus, M. hiemalis, P.* *chrysogenum,* and *T. koningii* were incubated on a malt medium (malt extract 20 g/L, dextrose 20 g/L, and peptone 1 g/L). Other microbes were cultured on a potato dextrose medium (24 g/L).

### 3.3. Isolation of Active Compounds from Broussonetia kazinoki

The root barks (650 g) of *B. kazinoki* were extracted with 94% ethanol (3 × 6 L) under sonication at room temperature. The combined ethanol extract was concentrated under reduced pressure, which was suspended in water and successively partitioned using hexane, dichloromethane (CH_2_Cl_2_), ethyl acetate (EtOAc), and butanol. The CH_2_Cl_2_ extract was subjected to silica gel column chromatography, using hexane:EtOAc mixtures to give sixteen fractions. Fraction 12 was then chromatographed using Sephadex LH-20 and eluted with methanol to give three subfractions. Subfraction 12-2 was purified by a C_18_ HPLC column with a methanol:water gradient elution system (75→80%) to afford compounds **1** (110 mg), **2** (8 mg), and **5** (4 mg). Fraction 14 was applied to Sephadex LH-20 column chromatography and eluted with methanol to give four subfractions. Subfraction 14-3 was further purified by semi-preparative HPLC with a methanol:water gradient elution system (75→80%) to afford compounds **3** (24 mg) and **4** (3 mg).

Kazinol C (**1**)

Oily substance. UV (MeOH) λ_max_: 224, 284 nm. ^1^H-NMR (DMSO-*d*_6_, 500 MHz) δ 6.70 (1H, s, H-6′), 6.43 (1H, s, H-2″), 6.28 (1H, s, H-3′), 6.19 (1H, dd, *J* = 10.8, 17.5 Hz, H-10′), 4.98 (1H, t, *J* = 6.6 Hz, H-8″), 4.87 (1H, dd, *J* = 1.6, 17.4 Hz, H-11′), 4.86 (1H, dd, *J* = 1.6, 10.7 Hz, H-11′), 4.86 (1H, overlapped, H-13″), 3.20 (2H, d, *J* = 6.5 Hz, H-7″), 3.08 (2H, d, *J* = 5.6 Hz, H-12″), 2.40 (2H, t, *J* = 7.3 Hz, H-1), 2.35 (2H, t, *J* = 7.5 Hz, H-3), 1.69 (2H, m, H-2), 1.66 (3H, s, H-15″), 1.63 (3H, s, H-10″), 1.61 (3H, s, H-11″), 1.60 (3H, s, H-16″), 1.35 (6H, s, H-8′/9′). ^13^C-NMR (DMSO-*d*_6_, 150 MHz) δ 154.4 (C-2′), 154.0 (C-4′), 148.9 (C-10′), 143.0 (C-3″), 141.4 (C-4″), 131.3 (C-9″), 130.0 (C-1″), 129.9 (C-14″), 128.6 (C-5″), 128.1 (C-6′), 127.3 (C-6″), 125.1 (C-5′), 124.5 (C-13″), 124.1 (C-8″), 118.1 (C-1′), 114.1 (C-2″), 109.6 (C-11′), 104.0 (C-3′), 39.8 (C-7′), 32.9 (C-3), 32.4 (C-2), 30.1 (C-1), 27.5 (C-7″), 27.4 (C-8′/9′), 25.9 (C-12″), 25.9 (C-11″), 25.7 (C-16″), 18.2 (C-10″), 18.2 (C-15″).

Kazinol E (**2**)

Oily substance. UV (MeOH) λ_max_: 224, 284 nm. ^1^H-NMR (CDCl_3_, 500 MHz) δ 6.94 (2H, s, H-5/2′), 6.39 (1H, s, H-8), 6.19 (1H, dd, *J* = 10.7, 17.7 Hz, H-22), 5.33 (1H, dd, *J* = 0.7, 17.7 Hz, H-23), 5.27 (2H, dd, *J* = 0.7, 10.7 Hz, H-23), 5.14 (1H, t, *J* = 6.6 Hz, H-10), 5.09 (1H, dd, *J* = 1.6, 10.7 Hz, H-2), 5.00 (1H, t, *J* = 5.6 Hz, H-15), 3.39 (2H, d, *J* = 6.6 Hz, H-9), 3.36 (1H, dd, *J* = 5.6, 16.5 Hz, H-14), 3.25 (1H, dd, *J* = 5.6, 16.5 Hz, H-14), 2.91 (1H, m, H-4), 2.75 (1H, m, H-4), 2.09 (1H, m, H-3), 1.97 (1H, m, H-3), 1.80 (3H, s, H-12), 1.73 (3H, s, H-13), 1.69 (3H, s, H-17), 1.67 (3H, s, H-18), 1.43 (6H, s, H-20/21).

Kazinol F (**3**)

Yellow powder. UV (MeOH) λ_max_: 224, 284 nm. ^1^H-NMR (DMSO-*d*_6_, 500 MHz) ^1^H-NMR (DMSO-*d*_6_, 500 MHz) δ 6.76 (1H, d, *J* = 8.1 Hz, H-6′), 6.42 (1H, s, H-2″), 6.25 (1H, d, *J* = 2.4 Hz, H-3′), 6.14 (1H, dd, *J* = 8.1, 2.4 Hz, H-5′), 4.99 (1H, t, *J* = 6.6 Hz, H-8″), 4.86 (1H, t, *J* = 6.2 Hz, H-13″), 3.21 (2H, d, *J* = 6.6 Hz, H-7‴), 3.08 (2H, d, *J* = 6.2 Hz, H-12‴), 2.42 (2H, t, *J*= 7.5 Hz, H-1), 2.35 (2H, t, *J* = 7.9 Hz, H-3), 1.66 (3H, s, H-10″), 1.64 (3H, s, H-15″), 1.63 (2H, overlapped, H-2), 1.61(6H, s, H-11″/16″). ^13^C-NMR (DMSO-*d*_6_, 150 MHz) δ 155.8 (C-4′), 155.6 (C-2′), 142.3 (C-3″), 140.9 (C-4″), 132.0 (C-1″), 130.0 (C-6′), 129.8 (C-14″), 129.7 (C-9″), 129.4 (C-5″), 127.1 (C-6″), 124.8 (C-13″), 123.9 (C-8″), 119.9 (C-1′), 113.4 (C-2″), 105.9 (C-5′), 102.1 (C-3′), 32.6 (C-3), 32.1 (C-2), 29.4 (C-1), 27.0 (C-7″), 25.1 (C-12″), 24.5 (C-16″), 24.5 (C-11″), 16.7 (C-15″), 16.6 (C-10″).

Broussonol N (**4**)

Yellow powder. ${\left[\alpha \right]}_{D}^{20}$: +74.6° (*c* 0.50, MeOH). UV (MeOH) λ_max_: 210, 291 nm. IR *ν*_max_: 3400, 2924, 1634, 1454, 1288, 1085, 837 cm^−1^. ^1^H-NMR (Acetone-*d*_6_, 600 MHz) δ 7.05 (1H, s, H-2′), 5.95 (1H, s, H-6), 5.89 (1H, s, H-8), 5.31 (1H, d, *J* = 11.5 Hz, H-2), 5.12 (1H, t, *J* = 6.6 Hz, H-10), 5.08 (1H, t, *J* = 6.1 Hz, H-15), 4.64 (1H, d, *J* = 11.5 Hz, H-3), 3.46 (1H, dd, *J* = 6.1, 16.4 Hz, H-14), 3.40 (2H, d, *J* = 6.2 Hz, H-9), 3.33 (1H, dd, *J* = 6.1, 16.4 Hz, H-14), 1.75 (3H, s, H-17), 1.66 (3H, s, H-13), 1.62 (3H, s, H-12), 1.61 (3H, s, H-18). ^13^C-NMR (Acetone-*d*_6_, 150 MHz) δ 198.0 (C-4), 169.7 (C-7), 165.0 (C-5), 164.4 (C-8a), 144.9 (C-4′), 143.4 (C-3′), 132.8 (C-6′), 131.1 (C-11), 130.9 (C-16), 127.8 (C-5′), 127.0 (C-1′), 125.5 (C-15), 124.7 (C-10), 113.1 (C-2′), 101.0 (C-4a), 97.5 (C-6), 96.5 (C-8), 80.9 (C-2), 73.0 (C-3), 27.9 (C-14), 26.2 (C-9), 25.9 (C-13), 25.8 (C-18), 18.2 (C-12), 18.2 (C-17). HRFDMS *m*/*z* 440.1841 [M]^+^ (calcd. for C_25_H_28_O_7_, 440.1835).

Kazinol X (**5**)

Oily substance. ${\left[\alpha \right]}_{D}^{20}$: −6.31° (*c* 0.19, MeOH). UV (MeOH) λ_max_: 228, 285 nm. IR *ν*_max_: 3320, 2941, 2832, 1449, 1022, 650 cm^−1^. ^1^H-NMR (CDCl_3_, 400 MHz) δ 6.93 (1H, s, H-6′), 6.61 (1H, s, H-2″), 6.32 (1H, s, H-3′), 6.17 (1H, dd, *J* = 10.6, 17.7 Hz, H-10′), 5.32 (1H, dd, *J* = 0.6, 17.7 Hz, H-11′), 5.26 (1H, dd, *J* = 0.6, 10.6 Hz, H-11′), 5.01 (1H, t, *J* = 6.6 Hz, H-13″), 4.73 (1H, t, *J* = 9.2 Hz, H-8″), 3.31 (3H, s, -OMe), 3.16 (2H, d, *J* = 6.6 Hz, H-12″), 3.09 (1H, dd, *J* = 9.6, 15.8 Hz, H-7″), 3.02 (1H, dd, *J* = 8.8, 15.8 Hz, H-7″), 2.59 (2H, m, H-1), 2.54 (2H, m, H-3), 1.81 (2H, m, H-2), 1.70 (3H, s, H-15″), 1.67 (3H, s, H-16″), 1.39 (6H, s, H-8′/9′), 1.24 (3H, s, H-11″), 1.20 (3H, s, H-10″). ^13^C-NMR (CDCl_3_, 100 MHz) δ 153.5 (C-2′), 153.2 (C-4′), 148.4 (C-10′), 144.2 (C-4″), 137.7 (C-3″), 133.3 (C-14″), 131.2 (C-5″), 127.6 (C-1″), 127.5 (C-6′), 126.9 (C-6″), 124.2 (C-5′), 122.9 (C-13″), 120.1 (C-1′), 116.0 (C-2″), 113.1 (C-11′), 104.7 (C-3′), 88.6 (C-8″), 76.4 (C-9″), 49.8 (-OMe), 39.8 (C-7′), 32.1 (C-3), 31.8 (C-2), 31.1 (C-7″), 29.6 (C-1), 28.8 (C-12″), 27.2 (C-8′,9′), 25.7 (C-16″), 20.8 (C-11″), 19.8 (C-10″), 17.9 (C-15″). HRFDMS *m*/*z* 494.3034 [M]^+^ (calcd. for C_31_H_42_O_5_, 494.3032).

### 3.4. Microbial Screening Procedures

The culture fermentation was carried out according to the usual two-stage procedure [24,25,26]. In the screening studies, the actively growing microbial cultures were inoculated in 250 mL flasks containing 50 mL of media and incubated in a temperature-controlled shaking incubator with gentle agitation (200 rpm) at 25 °C for one day. Then 100 μL of the prepared ethanol solution (10 mg/mL) of each substrate was added to the flask and further incubated for another seven days under the same condition. Sampling and TLC monitoring were performed at an interval of 24 h. Culture controls consisted of fermentation cultures in which the microorganisms were grown without the addition of substrates.

### 3.5. Scale-Up Fermentation of ***1*** and ***3*** with Mucor hiemalis

Scale-up fermentation was carried out with *M. hiemalis* using 500 mL flasks each containing 150 mL of malt media and 5 mg of compound **1** (total of 110 mg) under the same temperature-controlled shaking conditions for five days. After fermentation, the microbial cultures were extracted with the same volume of EtOAc three times and then the combined organic layers were concentrated in vacuo. The EtOAc extract of kazinol C (**1**) was separated by semi-preparative HPLC using isocratic 83% MeOH to afford metabolites **6** (4.6 mg), **7** (7.8 mg), **8** (3.5 mg), and **9** (4.5 mg). A similar fermentation process was performed for compound **3** (65 mg in total), which was incubated for three days. The yielded EtOAc extract was subjected to a C_18_ HPLC column using isocratic 77% MeOH under a flow rate of 2 mL/min to afford metabolites **10** (2.5 mg) and **11** (3.91 mg).

Kazinol C-3″-*O*-β-d-glucopyranoside (**6**)

Oily substance. UV (MeOH) λ_max_: 228, 285 nm. IR *ν*_max_: 3353, 2925, 1606, 1375, 1299, 1071, 598 cm^−1^. ^1^H-NMR (DMSO-*d*_6_, 600 MHz) δ 6.81 (1H, s, H-2″), 6.69 (1H, s, H-6′), 6.29 (1H, s, H-3′), 6.19(1H, dd, *J* = 10.8, 17.8 Hz, H-10′), 4.99 (1H, t, *J* = 6.5 Hz, H-8″), 4.87 (1H, d, *J* = 17.8 Hz, H-11′), 4.85 (1H, d, *J* = 10.8 Hz, H-11′), 4.84 (1H, overlapped, H-13″), 4.50 (1H, d, *J* = 7.1 Hz, H-1‴), 3.69 (1H, d, *J* = 11.5 Hz, H-6‴), 3.51(1H, m, H-6‴), 3.26 (1H, m, H-2‴/3‴), 3.25 (1H, m, H-5‴), 3.24 (2H, m, H-7″), 3.19 (1H, m, H-4‴), 3.12 (2H, d, *J* = 5.0 Hz, H-12″), 2.41 (2H, m, H-1), 2.40 (2H, m, H-3), 1.68 (3H, s, H-10″), 1.64 (2H, m, H-2), 1.63 (3H, s, H-15″), 1.62 (3H, s, H-11″), 1.60 (3H, s, H-16″), 1.34 (6H, s, H-8′/9′). ^13^C-NMR (DMSO-*d*_6_, 150 MHz) δ 154.0 (C-2′), 153.6 (C-4′), 148.4 (C-10′), 143.1 (C-3″), 142.9 (C-4″), 132.4 (C-6″), 131.0 (C-1″), 130.1 (C-14″), 130.0 (C-9″), 127.6 (C-6′), 126.7 (C-5″), 123.9 (C-13″), 123.6 (C-5′), 123.5 (C-8″), 117.5 (C-1′), 116.0 (C-2″), 109.2 (C-11′), 103.5 (C-3′), 103.4 (C-1‴), 77.1 (C-5‴), 75.8 (C-3‴), 73.4 (C-2‴), 69.7 (C-4‴), 60.7 (C-6‴), 39.4 (C-7′), 32.5 (C-3), 31.6 (C-2), 29.5 (C-1), 27.2 (C-12″), 26.9 (C-8′/9′), 25.5 (C-11″), 25.4 (C-16″), 25.2 (C-7″), 17.8 (C-10″), 17.8 (C-15″). HRFDMS *m*/*z* 626.3439 [M]^+^ (calcd. for C_36_H_50_O_9_, 626.3455).

Kazinol C-2′, 3″-di-*O*-β-d-glucopyranoside (**7**)

Oily substance. UV (MeOH) λ_max_: 228, 285 nm. IR *ν*_max_: 3349, 2926, 1598, 1413, 1071, 607 cm^−1^. ^1^H-NMR (DMSO-*d*_6_, 600 MHz) δ 6.82 (1H, s, H-2″), 6.77 (1H, s, H-6′), 6.60 (1H, s, H-3′), 6.21 (1H, dd, *J* = 10.8, 17.1 Hz, H-10′), 5.00 (1H, t, *J* = 6.4 Hz, H-8″), 4.89 (1H, d, *J* = 17.1 Hz, H-11′), 4.88 (1H, d, *J* = 10.8 Hz, H-11′), 4.84 (1H, t, *J* = 6.0 Hz, H-13″), 4.64 (1H, d, *J* = 6.5 Hz, H-1″″), 4.51 (1H, d, *J* = 6.6 Hz, H-1‴), 3.71 (1H, d, *J* = 11.3 Hz, H-6‴), 3.69 (1H, d, *J* = 11.2 Hz, H-6″″), 3.53 (2H, m, H-6‴/6″″), 3.27 (3H, m, H-2‴/3‴/5‴), 3.25 (2H, m, H-2″″/3″″), 3.24 (4H, m, H-7″), 3.21 (1H, m, H-4″″/5″″), 3.18 (1H, m, H-4‴), 3.14 (2H, d, *J* = 4.8 Hz, H-12″), 2.54 (2H, m, H-1), 2.44 (2H, t, *J* = 7.4 Hz, H-3), 1.68 (3H, s, H-10″), 1.66 (2H, m, H-2), 1.64 (3H, s, H-15″), 1.62(3H, s, H-11″), 1.60 (3H, s, H-16″), 1.37 (6H, s, H-8′/9′). ^13^C-NMR (DMSO-*d*_6_, 150 MHz) δ 154.2 (C-4′), 154.1 (C-2′), 148.0 (C-10′), 143.1 (C-4″), 142.9 (C-3″), 132.5 (C-1″), 131.1 (C-6″), 130.1 (C-14″), 129.9 (C-9″), 127.3 (C-6′), 126.7 (C-5″), 126.5 (C-5′), 124.0 (C-13″), 123.5 (C-8″), 120.3 (C-1′), 116.1 (C-2″), 109.5 (C-11′), 104.0 (C-3′), 103.5 (C-1‴), 101.5 (C-1″″), 77.1 (C-5‴), 76.9 (C-5″″), 76.8 (C-3″″), 75.8 (C-3‴), 73.5 (C-2‴), 73.4 (C-2″″), 69.7 (C-4‴), 69.6 (C-4″″), 60.7 (C-6‴/6″″), 39.5 (C-7′), 32.4 (C-3), 31.7 (C-2), 29.2 (C-1), 27.3 (C-12″), 26.7 (C-8′/9′), 25.5 (C-11″), 25.5 (C-16″), 25.2 (C-7″), 17.9 (C-15″), 17.8 (C-10″). HRFDMS *m*/*z* 788.3997 [M]^+^ (calcd. for C_42_H_60_O_14_, 788.3983).

Kazinol C-4″-*O*-β-d-glucopyranoside (**8**)

Oily substance. UV (MeOH) λ_max_: 228, 285 nm. IR *ν*_max_: 3363, 2925, 1596, 1378, 1069, 609 cm^−1^. ^1^H-NMR (DMSO-*d*_6_, 600 MHz δ 6.70 (1H, s, H-6′), 6.51 (1H, s, H-2″), 6.30 (1H, s, H-3′), 6.19 (1H, dd, *J* = 10.5, 17.5 Hz, H-10′), 4.98 (1H, t, *J* = 5.8 Hz, H-8″), 4.88 (1H, d, *J* = 17.0 Hz, H-11′), 4.86 (1H, d, *J* = 9.6 Hz, H-11′), 4.86 (1H, overlapped, H-13″), 4.42 (1H, d, *J* = 7.6 Hz, H-1‴), 3.64 (1H, d, *J* = 11.9 Hz, H-6‴), 3.16 (1H, m, H-7″), 3.49(1H, dd, *J* = 4.4, 11.9 Hz, H-6‴), 3.27 (1H, m, H-2‴), 3.25 (1H, m, H-7″), 3.24 (1H, m, H-3‴), 3.17 (1H, m, H-4‴), 3.15 (1H, m, H-5‴), 3.09 (2H, m, H-12″), 2.42 (2H, m, H-1), 2.39 (2H, m, H-3), 1.65 (3H, s, H-10″), 1.63 (5H, s, H-15″/2), 1.61(6H, s, H-11″/16″), 1.35 (6H, s, H-8′/9′). ^13^C-NMR (DMSO-*d*_6_, 150 MHz) δ 154.0 (C-2′), 153.6 (C-4′), 148.4 (C-10′), 147.0 (C-3″), 141.9 (C-4″), 137.9 (C-1″), 133.9 (C-5″), 130.0 (C-14″), 129.4 (C-9″), 128.5 (C-6″), 127.6 (C-6′), 124.3 (C-8″), 124.1 (C-13″), 123.6 (C-5′), 117.4 (C-1′), 114.9 (C-2″), 109.2 (C-11′), 105.9 (C-1‴), 103.6 (C-3′), 77.3 (C-5‴), 76.2 (C-3‴), 74.1 (C-2‴), 69.5 (C-4‴), 60.8 (C-6‴), 39.2 (C-7′), 32.4 (C-3), 31.5 (C-2), 29.6 (C-1), 27.1 (C-12″), 26.9 (C-8′/9′), 25.7 (C-7″), 25.4 (C-16″/11″), 17.8 (C-10″), 17.7 (C-15″). HRFDMS *m*/*z* 626.3469 [M]^+^ (calcd. for C_36_H_50_O_9_, 626.3455).

Kazinol C-3″, 4″-di-*O*-β-d-glucopyranoside (**9**)

Oily substance. UV (MeOH) λ_max_: 228, 285 nm. IR *ν*_max_: 3342, 2926, 1597, 1397, 1071, 611 cm^−1^. ^1^H-NMR (DMSO-*d*_6_, 600 MHz) δ 6.93 (1H, s, H-2″), 6.70 (1H, s, H-6′), 6.29 (1H, s, H-3′), 6.19 (1H, dd, *J* = 10.7, 17.6 Hz, H-10′), 5.07 (1H, t, *J* = 6.8 Hz, H-8″), 4.87 (1H, d, *J* = 17.4 Hz, H-11′), 4.85 (1H, d, *J* = 10.4 Hz, H-11′), 4.85 (1H, d, *J* = 7.4 Hz, H-1″″), 4.84 (1H, overlapped, H-13″), 4.65 (1H, d, *J* = 7.6 Hz, H-1‴), 3.69 (1H, d, *J* = 10.4 Hz, H-6‴), 3.63 (1H, d, *J* = 11.4 Hz, H-6″″), 3.59 (1H, m, H-7″), 3.51 (1H, m, H-6‴), 3.41 (1H, overlapped, H-6″″), 3.31 (1H, m, H-2″″), 3.28 (1H, m, H-5″″), 3.26 (1H, m, H-3‴), 3.23 (2H, m, H-2‴/3″″), 3.19 (1H, m, H-4″″), 3.17 (1H, m, H-7″), 3.11 (2H, m, H-12″), 3.09 (1H, m, H-4‴), 3.02 (1H, m, H-5‴), 2.42 (4H, m, H-1/3), 1.67 (2H, m, H-2), 1.66 (3H, s, H-10″), 1.60 (9H, s, H-11″/15″/16″), 1.34 (6H, s, H-8′/9′). ^13^C-NMR (DMSO-*d*_6_, 150 MHz) δ 154.0 (C-4′), 153.6 (C-2′), 148.4 (C-10′), 147.7 (C-3″), 142.2 (C-4″), 137.1 (C-1″), 134.5 (C-5″), 131.9 (C-6″), 130.3 (C-14″), 129.4 (C-9″), 127.7 (C-6′), 124.0 (C-8″), 123.7 (C-13″), 123.7 (C-5′), 117.4 (C-1′), 115.4 (C-2″), 109.2 (C-11′), 103.6 (C-3′), 103.5 (C-1″″), 102.3 (C-1‴), 77.1 (C-5‴), 77.1 (C-5″″), 76.5 (C-3″″), 75.8 (C-3‴), 74.3 (C-2‴), 73.5 (C-2″″), 70.0 (C-4‴), 69.8 (C-4″″), 61.1 (C-6″″), 60.7 (C-6‴), 39.1 (C-7′), 32.5 (C-3), 31.2 (C-2), 29.5 (C-1), 27.2 (C-12″), 26.9 (C-8′/9′), 26.0 (C-7″), 25.4 (C-11″), 25.4 (C-16″), 17.9 (C-10″), 17.8 (C-15″). HRFDMS *m*/*z* 788.4016 [M]^+^ (calcd. for C_42_H_60_O_14_, 788.3983).

Kazinol Y (**10**)

Yellow powder. ${\left[\alpha \right]}_{D}^{20}$: −12.38 (*c* 0.42, MeOH). UV (MeOH) λ_max_: 228, 285 nm. IR *ν*_max_: 3354, 2921, 1591, 1457, 1378, 1269, 1096, 577 cm^−1^. ^1^H-NMR (DMSO-*d*_6_, 600 MHz) δ 6.76 (1H, d, *J* = 8.2 Hz, H-6′), 6.38 (1H, s, H-2″), 6.26 (1H, d, *J* = 2.3 Hz, H-3′), 6.10 (1H, dd, *J* = 2.4, 8.1 Hz, H-5′), 4.94 (1H, t, *J* = 6.7 Hz, H-13″), 4.58 (1H, t, *J* = 9.1 Hz, H-8″), 3.16 (3H, s, -OMe), 3.07 (2H, d, *J* = 6.8 Hz, H-12″), 2.97 (2H, dd, *J* = 4.3, 9.3 Hz, H-7″), 2.42 (2H, t, *J* = 7.4 Hz, H-1), 2.36 (2H, m, H-3), 1.66 (3H, s, H-15″), 1.62 (3H, s, H-16″), 1.61 (2H, m, H-2), 1.14(3H, s, H-11″), 1.10 (3H, s, H-10″). ^13^C-NMR (DMSO-*d*_6_, 150 MHz) δ 158.2 (C-4′), 157.8 (C-2′), 146.5 (C-4″), 140.7 (C-3″), 134.1 (C-14″), 132.0 (C-6″), 131.8 (C-6′), 129.3 (C-5″), 127.5 (C-1″), 125.2 (C-13″), 120.5 (C-1′), 118.1 (C-2″), 107.7 (C-5′), 104.4 (C-3′), 89.3 (C-8″), 77.5 (C-9″), 51.2 (-OMe), 34.0 (C-2), 33.7 (C-3), 32.1 (C-7″), 31.2 (C-1), 30.2 (C-12″), 27.4 (C-16″), 23.3 (C-11″), 21.7 (C-10″), 19.7 (C-15″). HRFDMS *m*/*z* 426.2414 [M]^+^ (calcd. for C_26_H_34_O_5_, 426.2406).

Kazinol F-4″-*O*-β-d-glucopyranoside (**11**)

Oily substance. UV (MeOH) λ_max_: 228, 285 nm. IR *ν*_max_: 3337, 2925, 1597, 1461, 1069, 608 cm^−1^. ^1^H-NMR (DMSO-*d*_6_, 600 MHz) δ 6.77 (1H, d, *J* = 8.2 Hz, H-6′), 6.50 (1H, s, H-2″), 6.25 (1H, d, *J* = 2.3Hz, H-3′), 6.11–6.10 (1H, dd, *J* = 2.3, 8.2 Hz, H-5′), 4.98 (1H, t, *J* = 6.5 Hz, H-8″), 4.87 (1H, t, *J* = 6.0 Hz, H-13″), 4.43 (1H, d, *J* = 7.7 Hz, H-1‴), 3.67 (1H, dd, *J* = 6.7, 14.6 Hz, H-7″), 3.65 (1H, d, *J* = 11.6 Hz, H-6‴), 3.49 (1H, d, *J* = 11.6 Hz, H-6‴), 3.28 (1H, m, H-2‴), 3.26 (1H, m, H-7″), 3.24 (1H, m, H-3‴), 3.17 (1H, m, H-4″), 3.16 (1H, m, H-5‴), 3.09 (2H, m, H-12″), 2.43 (2H, t, *J* = 7.1 Hz, H-1), 2.39 (2H, m, H-3), 1.65 (2H, s, H-10″), 1.64 (2H, overlapped, H-2), 1.63 (4H, s, H-15″), 1.61 (3H, s, H-11″), 1.60 (3H, s, H-16″). ^13^C-NMR (DMSO-*d*_6_, 150 MHz) δ 156.2 (C-4′), 155.7 (C-2′), 147.1 (C-3″), 141.9 (C-4″), 137.8 (C-1″), 133.9 (C-5″), 130.1 (C-14″), 129.8 (C-6′), 129.4 (C-9″), 128.5 (C-6″), 124.3 (C-8″), 124.1 (C-13″), 118.4 (C-1′), 114.8 (C-2″), 105.9 (C-1‴), 105.8 (C-5′), 102.4 (C-3′), 77.3 (C-5‴), 76.2 (C-3‴), 74.1 (C-2‴), 69.6 (C-4‴), 60.8 (C-6‴), 32.3 (C-3), 31.3 (C-2), 29.3 (C-1), 27.0 (C-12″), 25.7 (C-7″), 25.4 (C-11″), 25.4 (C-16″), 17.8 (C-10″), 17.7 (C-15″). HRFDMS *m*/*z* 558.2839 [M]^+^ (calcd. for C_31_H_42_O_9_, 558.2829).

### 3.6. Mushroom Tyrosinase Activity

The mushroom tyrosinase (Sigma, T3824) inhibition by compounds **1**–**11** was performed using the previously described method [10] with some modifications. All the compounds were first dissolved in dimethyl sulfoxide (DMSO) and then diluted into different concentrations (1, 2, 4, 10, 40, 80, and 160 µM). An amount of 100 µL of each sample solution and 50 µL of the 2 mM L-tyrosine solution in 0.1 M phosphate buffer (pH 6.5) were added to 96-well microplates. After incubation at room temperature for 5 min, 50 µL of mushroom tyrosinase (300 U/mL in phosphate buffer) was added to each well. After further incubation for 30 min under 37 °C, the absorbance of dopachrome produced in the mixture was determined at 490 nm with a microplate reader (SpectraMax 190; Molecular Devices, Sunnyvale, CA, USA). The percent of inhibition of tyrosinase activity was calculated using the formula: % inhibition = (A − B)/A × 100, in which A is the absorbance at 490 nm without the test sample, and B is the absorbance at 490 nm with the test sample. IC_50_ values were calculated from the mean values of data from four determinations.

### 3.7. Cytotoxic Activity

The cytotoxic activities of all the compounds (**1**–**11**) were evaluated using the MTT assay [24,28]. Briefly, the A375P (human melanoma), B16F10 and B16F1 (mouse melanoma) cell lines obtained from the Korean Cell Line Bank (Seoul, Korea) were cultured in Dulbecco’s Modified Eagle’s Medium (DMEM) (Gibco, CA, USA) containing penicillin (100 units/mL)-streptomycin (100 µg/mL) (Gibco, CA, USA) and 5% heat-inactivated fetal bovine serum (FBS) (Welgene, Korea) in a humidified chamber with 5% CO_2_ at 37 °C. The cells were seeded in 96-well plates at a density of 6 × 10^3^ cells/well and incubated for 24 h. The medium was then aspirated and replaced with 100 μL of fresh medium containing various concentrations of the test compound. After incubation for a further 24 h, the compound containing the medium was replaced with 100 μL of a MTT solution (0.5 mg/mL) and incubated for 4 h. Then the insoluble formazan crystals were dissolved in 100 µL of DMSO and measured at 490 nm on a microplate reader.

## 4. Conclusions

The activity-guided fractionation of the root barks of *B. kazinoki* led to the isolation of five compounds, including two previously unreported prenylated polyphenols **4** and **5**. The subsequent microbial transformation studies on the two major compounds, kazinol C (**1**) and kazinol F (**3**), resulted in the production of five glucosylated and one oxidized metabolite (**6**–**11**). All the obtained compounds were evaluated for their tyrosinase inhibitory and cytotoxic activities. Compounds **3**, **4**, **10** and **11**, which lack a prenyl group in the ring A of their structures, exhibited potent tyrosinase inhibitory activities, with glucosylated metabolite **11** being the most active. Although all the metabolites showed weaker cytotoxic activities than their parent compounds, the compound 8, which had a glucose moiety in its structure, exhibited a moderate activity against the cancer cell lines tested. All of these indicated that glycosylation plays a role in the biological activities of the prenylated polyphenols from *B. kazinoki*.

## Figures and Tables

**Table 1:** Tyrosinase inhibitory and cytotoxic effects of compounds **1**–**12** (IC_50_, µM).

Compound	Anti-Tyrosinase	Cell Line		
		A375P	B16F10	B16F1
**1**	>80	12.13 ± 0.24	26.01 ± 0.33	17.57 ± 0.33
**2**	>80	11.46 ± 0.24	12.13 ± 0.87	13.35 ± 0.22
**3**	2.12 ± 0.21	18.16 ± 0.09	19.89 ± 0.73	13.77 ± 0.88
**4**	24.11 ± 0.30	13.77 ± 1.00	27.79 ± 0.63	24.38 ± 1.82
**5**	>80	45.87 ± 0.65	43.24 ± 0.71	17.32 ± 0.22
**6**	>80	>80	>80	>80
**7**	>80	23.63 ± 0.52	24.22 ± 1.26	17.80 ± 1.65
**8**	>80	>80	>80	>80
**9**	>80	>80	>80	>80
**10**	3.36 ± 0.21	>80	>80	>80
**11**	0.71 ± 0.01	>80	>80	>80
Kojic acid	42.67 ± 1.44	-	-	-
5-FU	-	13.13 ± 1.55	4.61 ± 0.16	12.82 ± 0.16

Each value represents the mean ± SD.

## Data Availability

Not applicable.

## Supplementary Materials

The following are available online https://www.mdpi.com/article/10.3390/molecules27061879/s1. Table S1. Screening for the microorganisms that metabolize kazinols C (**1**) and F (**3**).; Figures S1–S53: NMR and HRFDMS spectra of compounds **1**–**11**.

## Sample Availability

Samples of the compounds are not available from the authors.
