# Supplementary material for: Microbial Transformation and Biological Activities of the Prenylated Aromatic Compounds from Broussonetia kazinoki
Source: Molecules. 2022 Mar 14;27(6):1879. doi: 10.3390/molecules27061879 (PMC8954733; doi:10.3390/molecules27061879)
Supplement: Supplementary file 1 [file molecules-27-01879-s001.zip › molecules-1622589-supplementary.pdf]

molecules

MDPI

Article

Microbial Transformation and Biological Activities of the Prenylated Aromatic Compounds from *Broussonetia kazinoki*

EunA Choi, Fubo Han, Jisu Park † and Ik-Soo Lee *

College of Pharmacy, Chonnam National University, Gwangju 61186, Korea; 206412@jnu.ac.kr (E.C.); hanfubo0306@gmail.com (F.H.); qkrwltn9410@naver.com (J.P.)
* Correspondence: islee@chonnam.ac.kr; Tel.: +82-62-530-2932
† Current address: Advanced Radiation Technology Institute, Korea Atomic Energy Research Institute, Jeongeup-si 56212, Korea

Table S1. Screening for the microorganisms that metabolize kazinols C (**1**) and F (**3**).

Microorganism	Capability*	
	Kazinol C	Kazinol F
A. alternata	(-)	(+)
A. coerulea	(-)	(+)
A. fumigatus	(-)	(-)
A. oryzae	(-)	(-)
C. elegans var. *elegans*	(-)	(-)
F. merismoides	(-)	(-)
G. deliquescens	(+)	(+)
G. cingulata	(-)	(-)
H. resinae	(-)	(-)
M. rubber	(-)	(-)
M. ramanniana var. *angulispora*	(-)	(-)
M. hiemalis	(++)	(++)
P. chrysogenum	(-)	(-)
T. koningii	(-)	(-)

* Capability of transformation Kazinols C (**1**) and F (**3**): (-) no transformation; (+) lower yield of metabolites; (++) higher yield of metabolites.

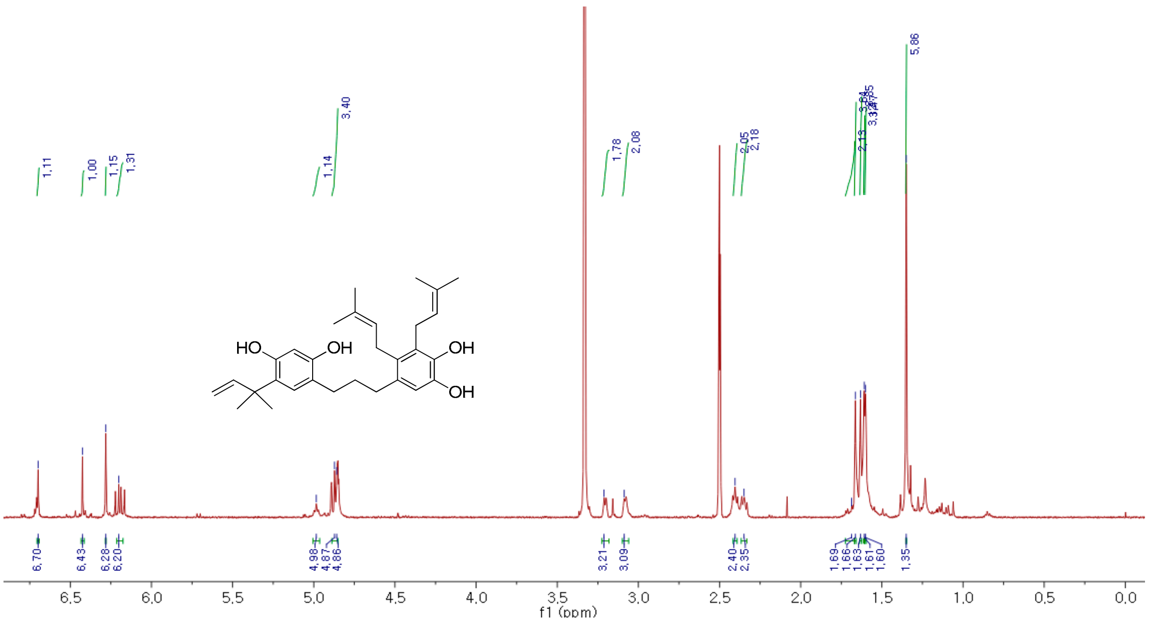

Figure S1. ^{1}H NMR spectrum of compound **1** recorded at 500 MHz in DMSO-*d*6.

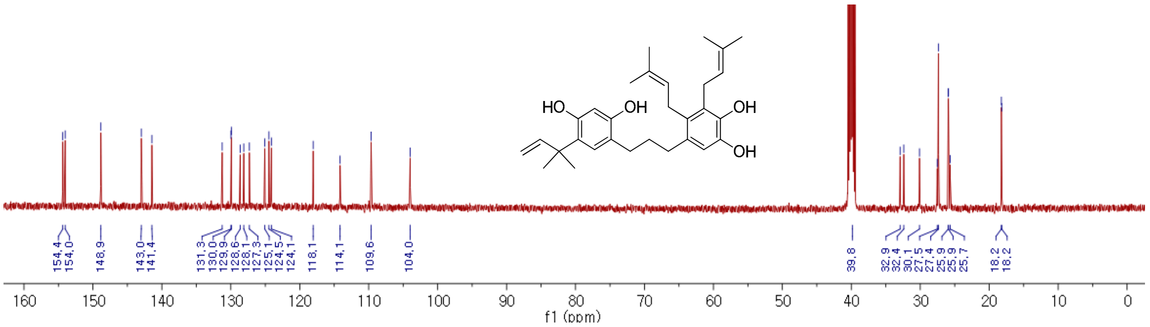

Figure S2. ^{13}C NMR spectrum of compound **1** recorded at 125 MHz in DMSO-*d*6.

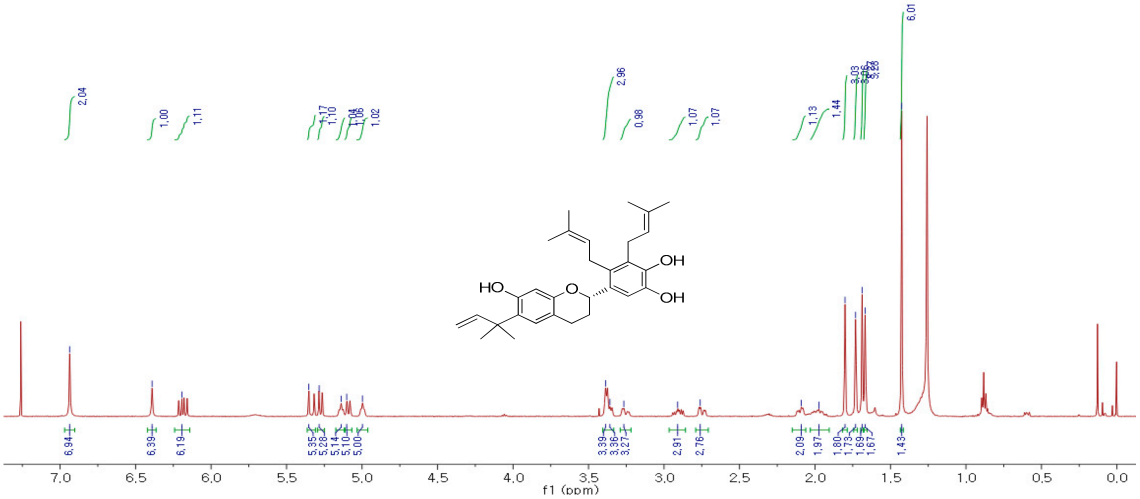

Figure S3. ^{1}H NMR spectrum of compound **2** recorded at 500 MHz in $CDCl_3$.

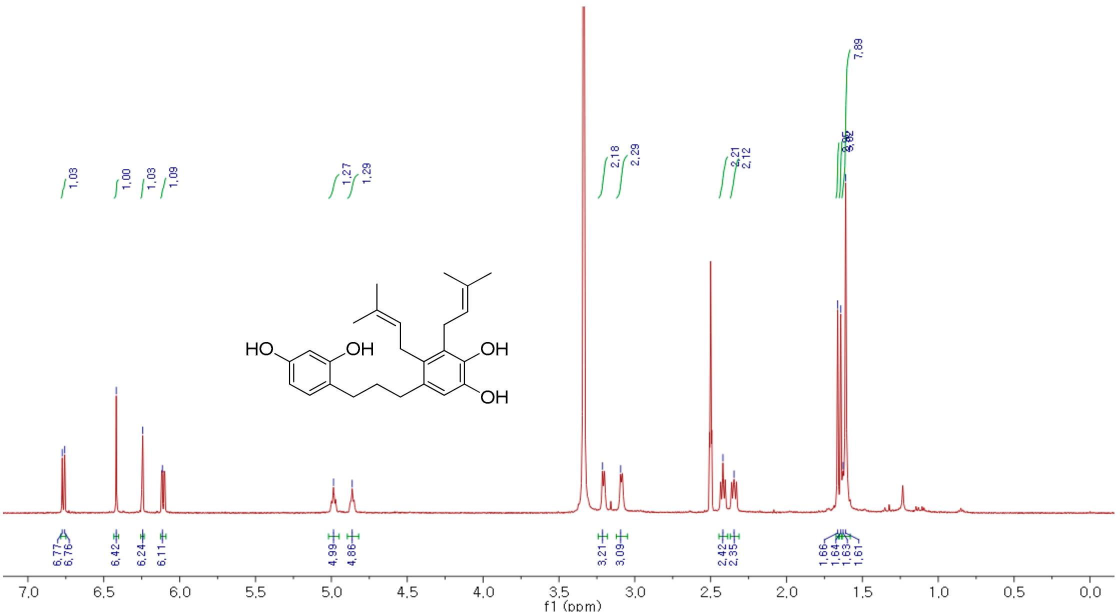

Figure S4. ^{1}H NMR spectrum of compound **3** recorded at 500 MHz in DMSO-*d*$_6$.

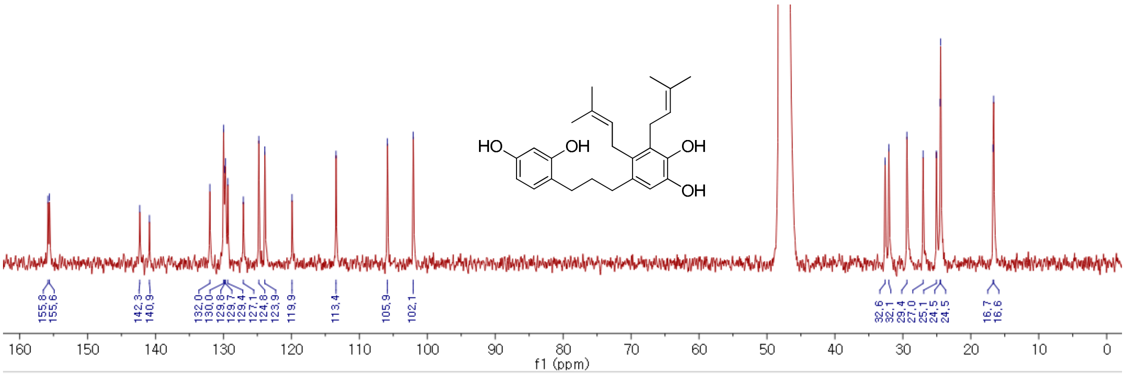

Figure S5. ^{13}C NMR spectrum of compound **3** recorded at 125 MHz in DMSO-*d*$_6$.

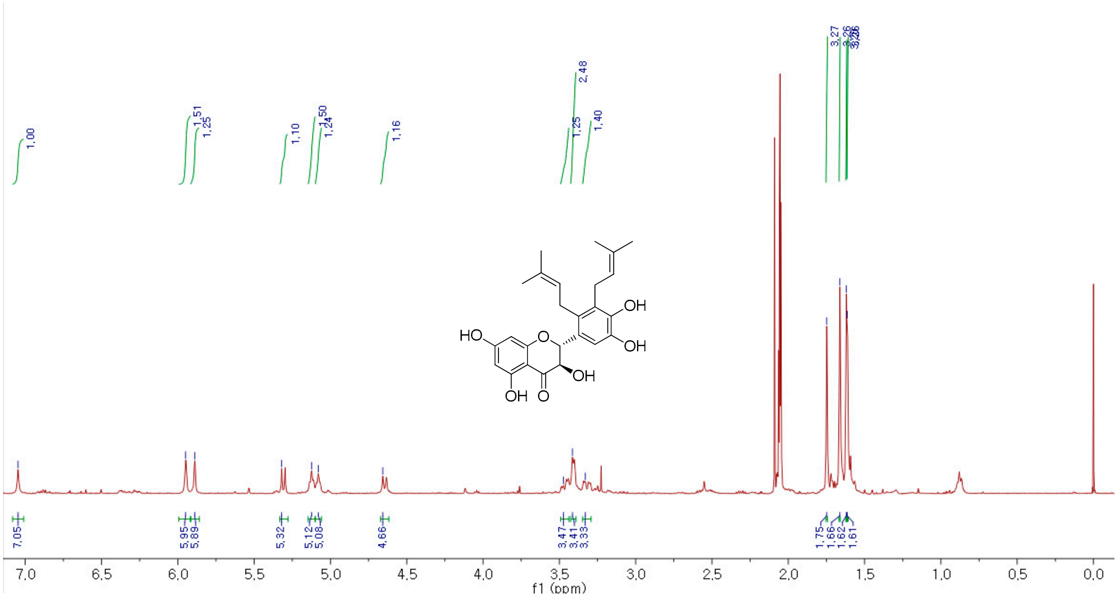

Figure S6. ^{1}H NMR spectrum of compound **4** recorded at 600 MHz in Acetone-*d*$_6$.

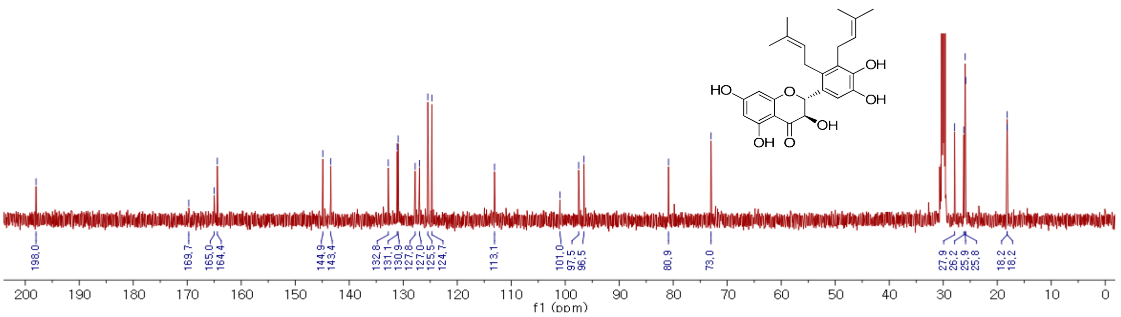

Figure S7. ^{13}C NMR spectrum of compound **4** recorded at 150 MHz in Acetone-*d*6.

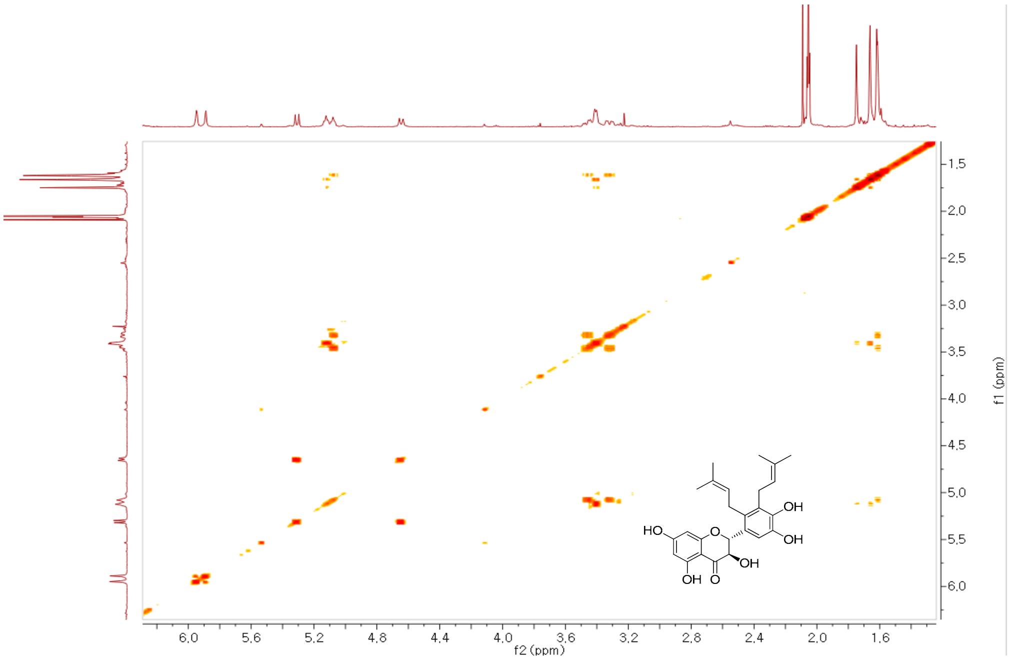

Figure S8. COSY spectrum of compound **4** recorded in Acetone-*d*6.

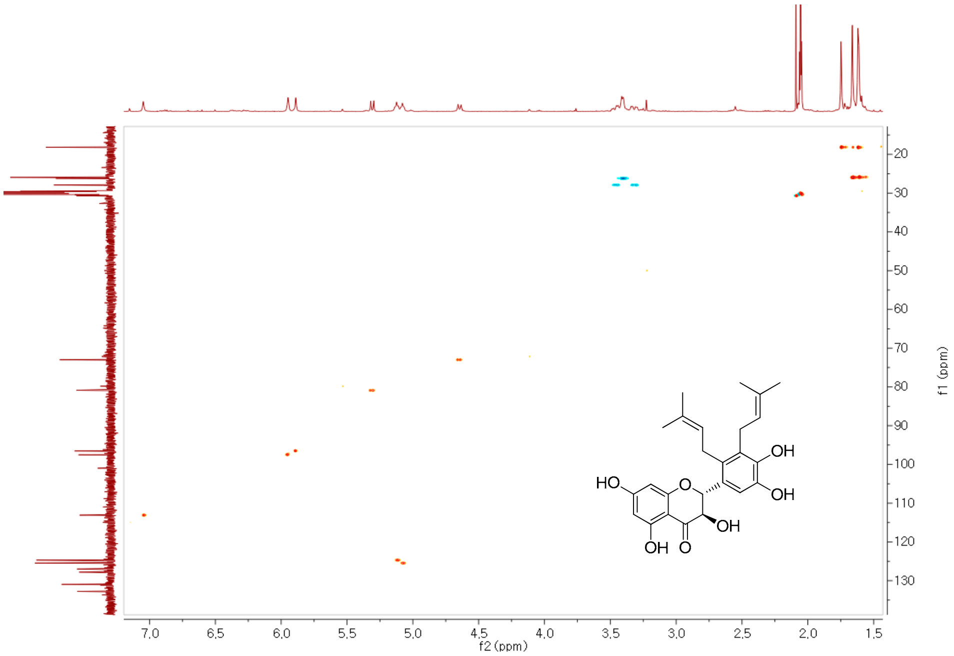

Figure S9. HSQC spectrum of compound **4** recorded in Acetone-*d*6.

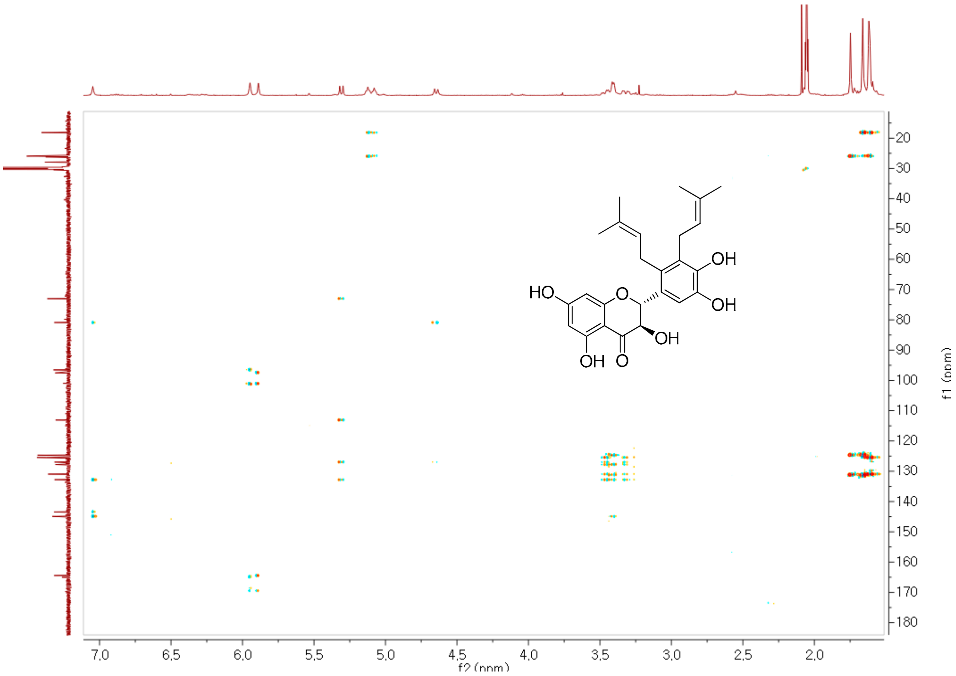

Figure S10. HMBC spectrum of compound **4** recorded in Acetone-d_6.

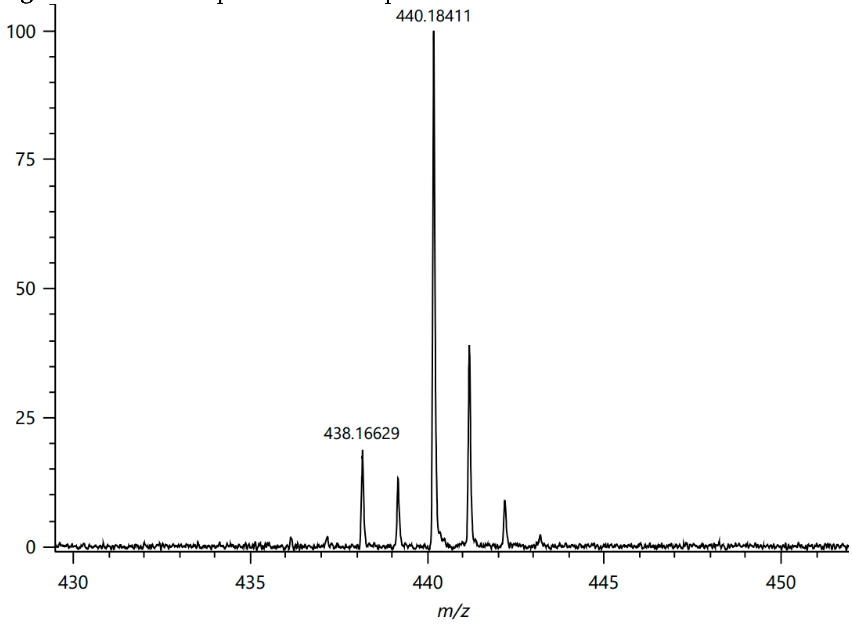

Figure S11. The HRFDMS spectrum of compound **4**.

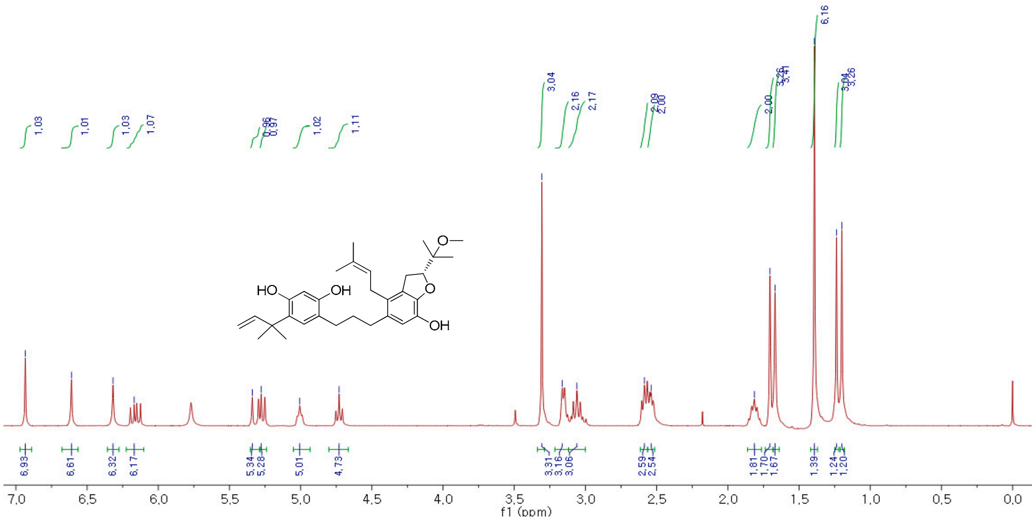

Figure S12. ^{1}H NMR spectrum of compound **5** recorded at 400 MHz in CDCl3.

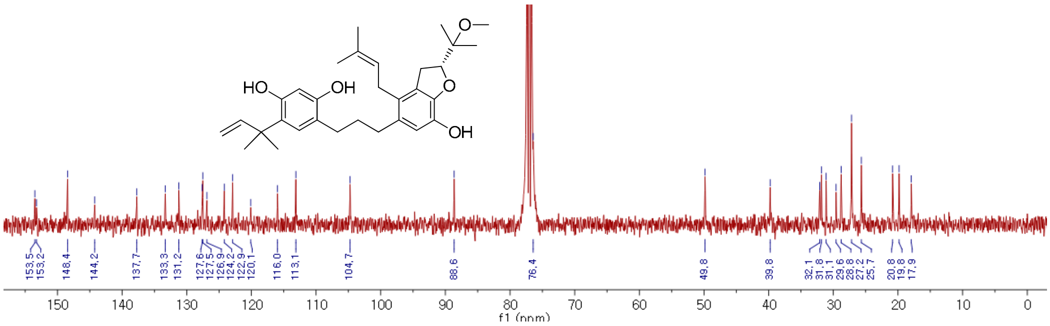

Figure S13. ^{13}C NMR spectrum of compound **5** recorded at 100 MHz in CDCl3.

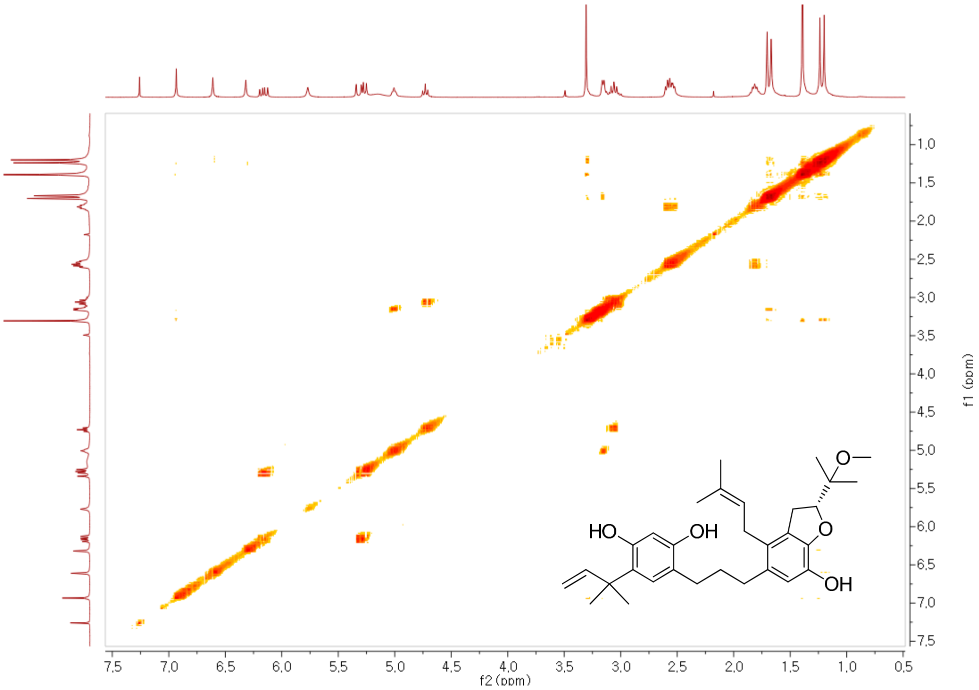

Figure S14. COSY spectrum of compound **5** recorded in CDCl3.

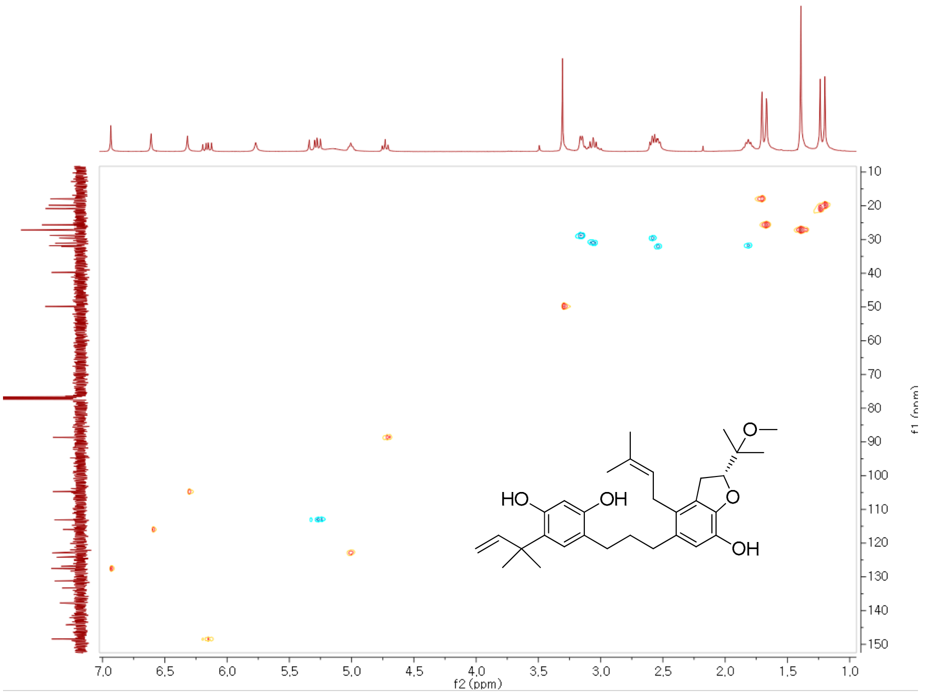

Figure S15. HSQC spectrum of compound **5** recorded in CDCl3.

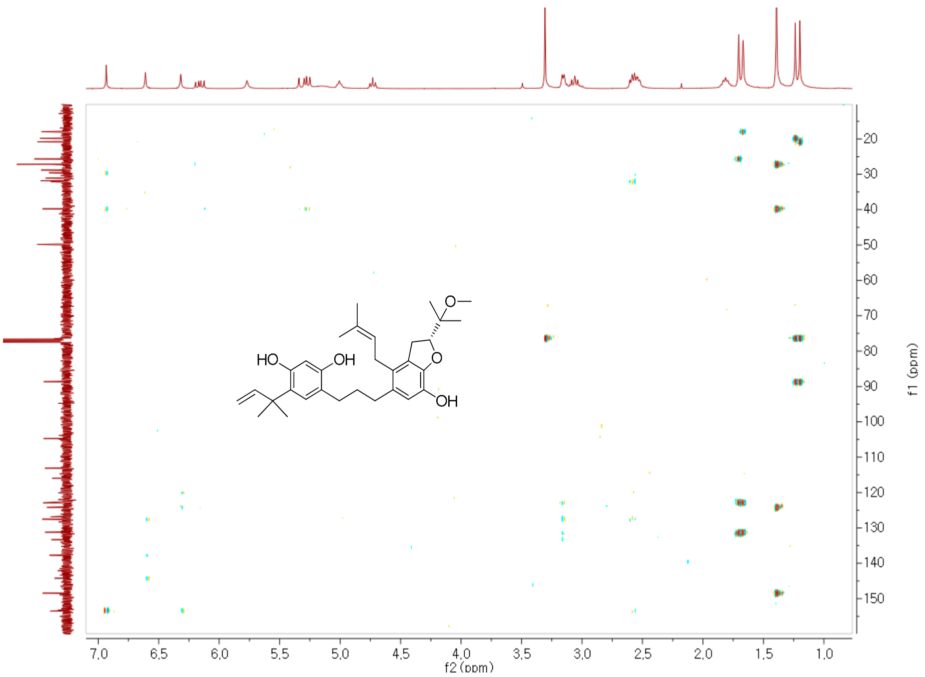

Figure S16. HMBC spectrum of compound **5** recorded in CDCl3.

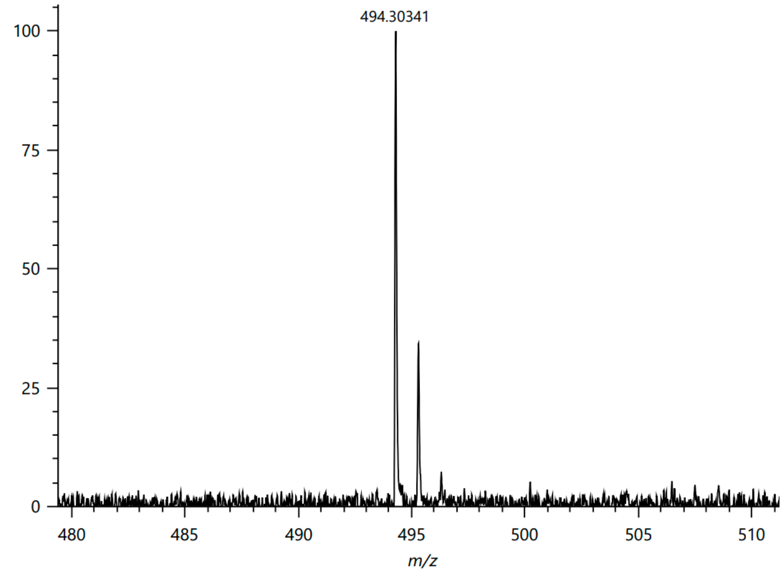

Figure S17. The HRFDMS spectrum of compound **5**.

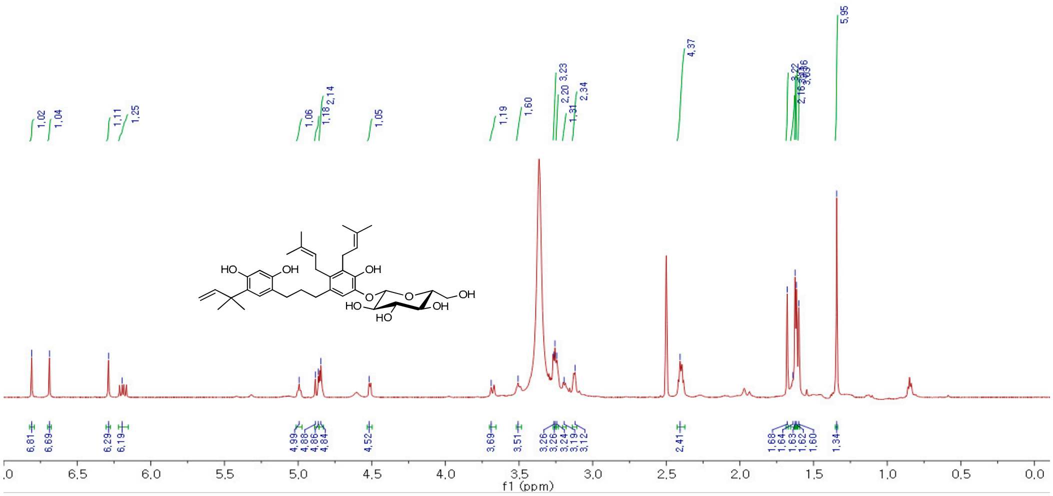

Figure S18. ^{1}H NMR spectrum of compound **6** recorded at 600 MHz in DMSO-d_6.

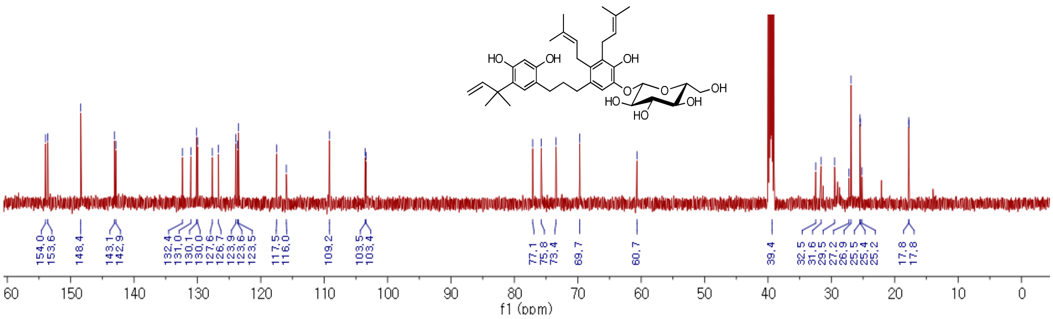

Figure S19. ^{13}C NMR spectrum of compound **6** recorded at 150 MHz in DMSO-d_6.

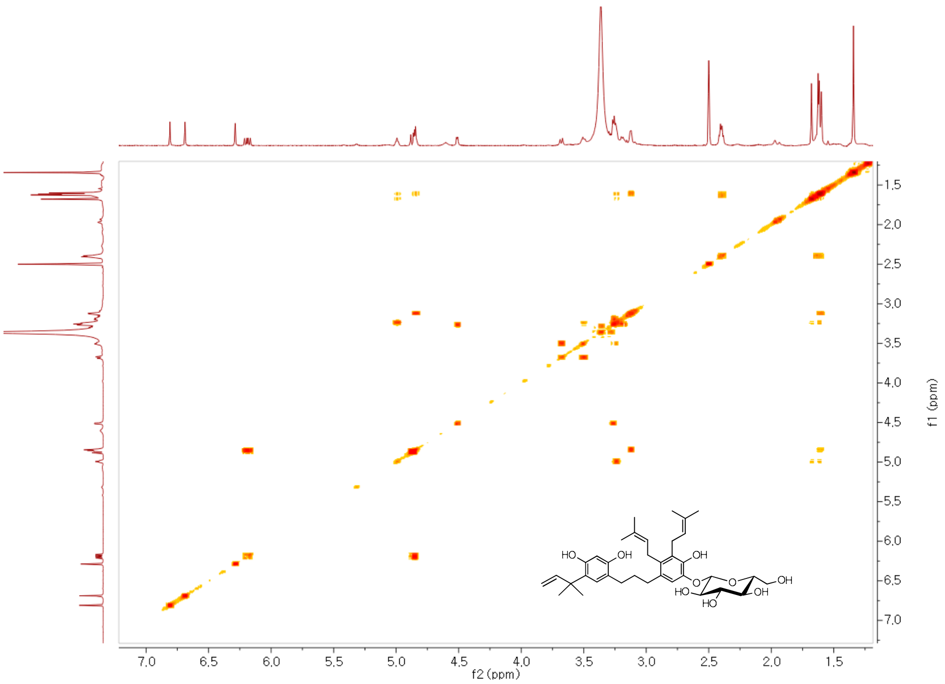

Figure S20. COSY spectrum of compound **6** recorded in DMSO-d_6.

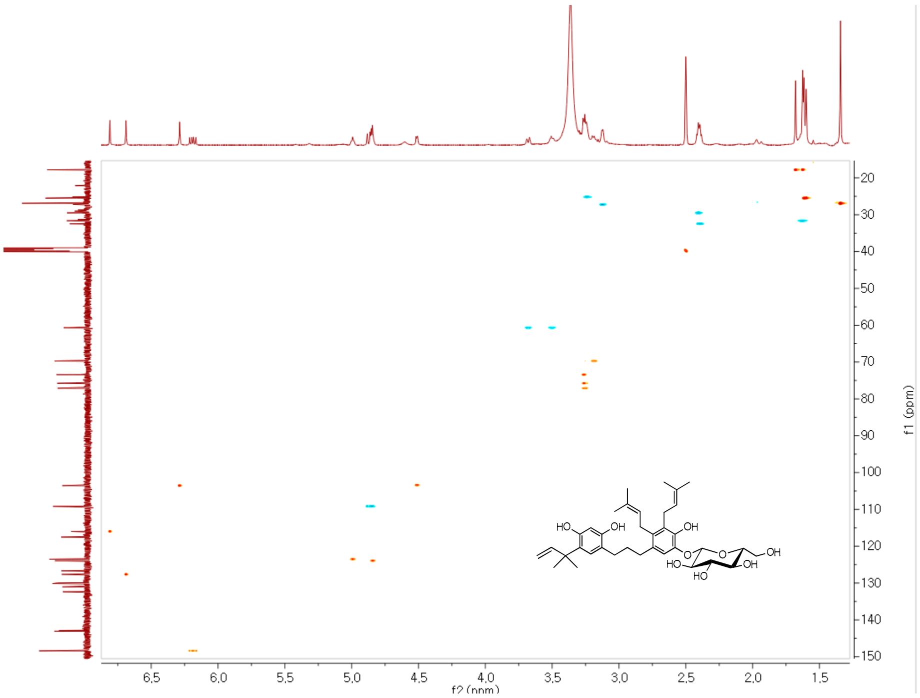

Figure S21. HSQC spectrum of compound **6** recorded in DMSO-d_6.

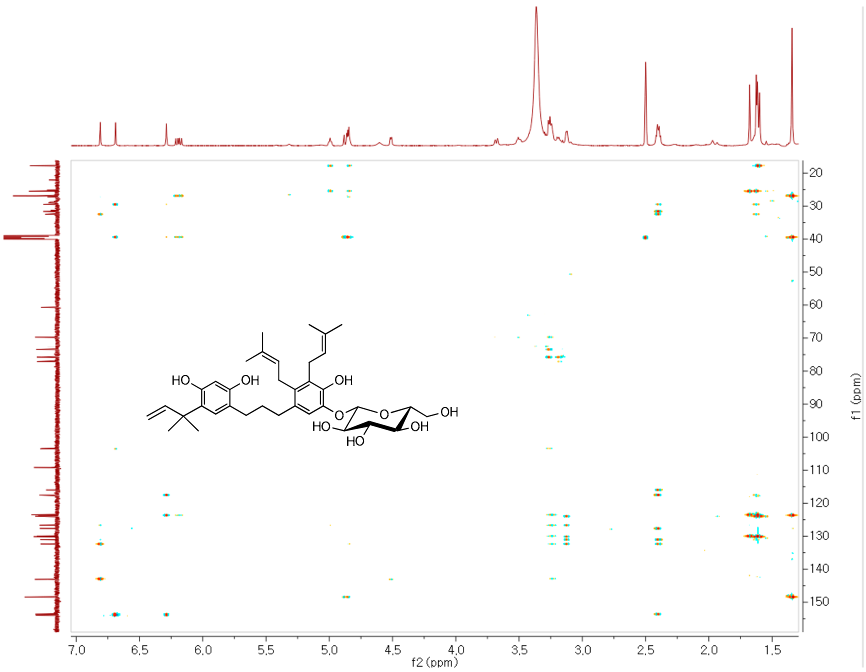

Figure S22. HMBC spectrum of compound **6** recorded in DMSO-d_6.

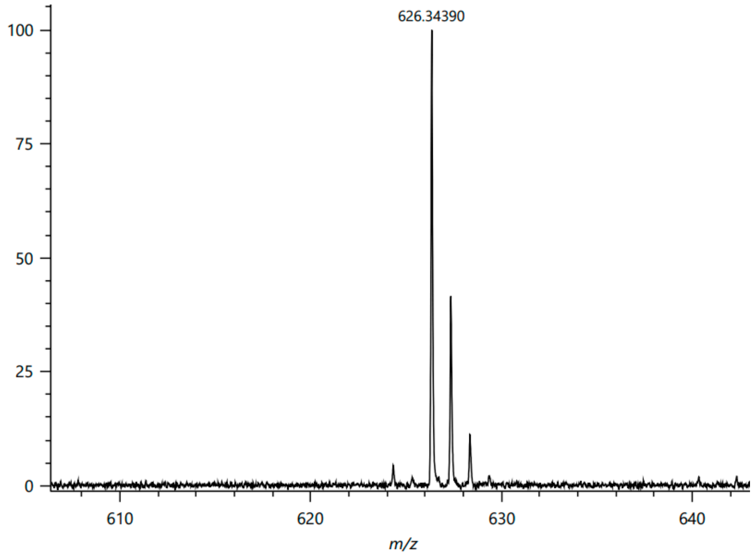

Figure S23. The HRFDMS spectrum of compound **6**.

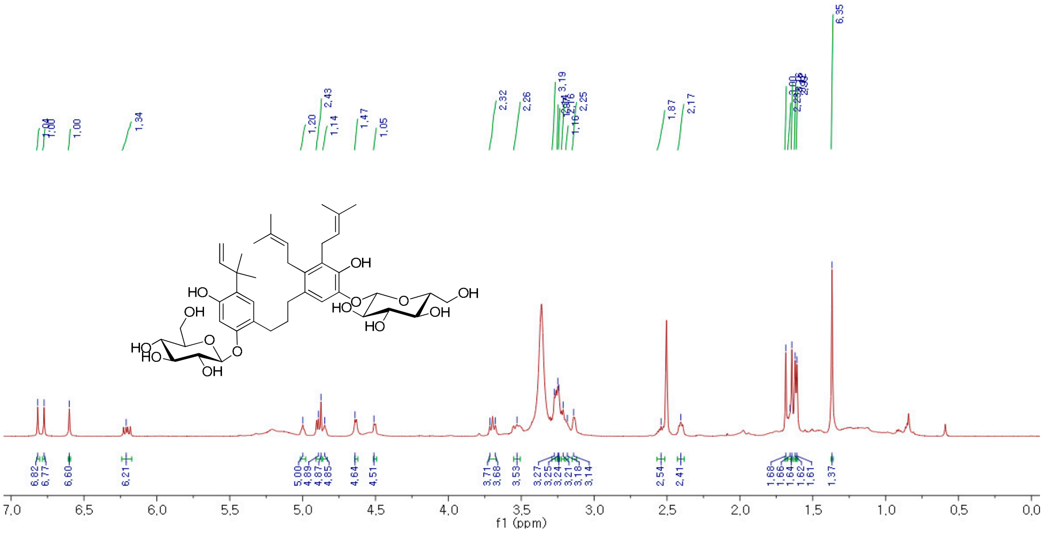

Figure S24. ^{1}H NMR spectrum of compound **7** recorded at 600 MHz in DMSO-*d*6.

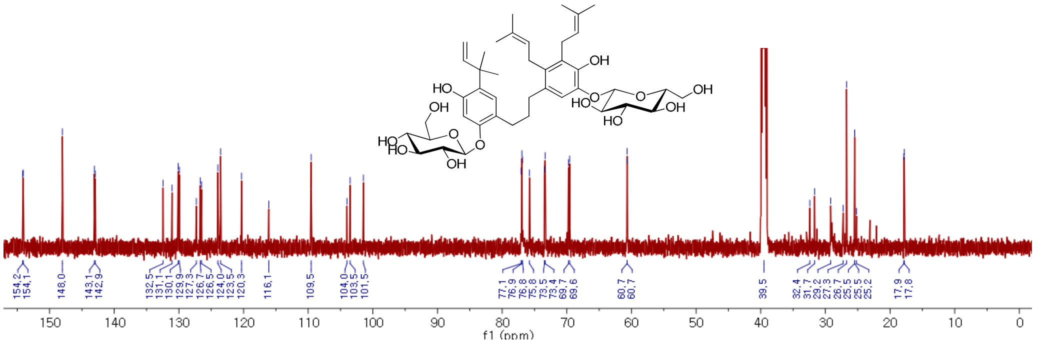

Figure S25. ^{13}C NMR spectrum of compound **7** recorded at 150 MHz in DMSO-*d*6.

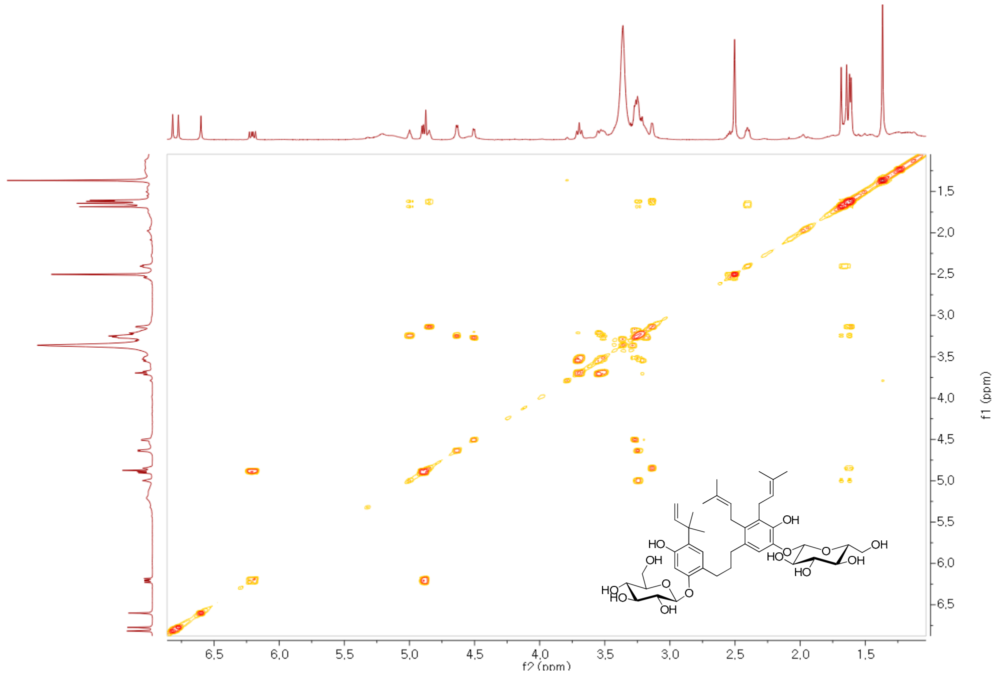

Figure S26. COSY spectrum of compound **7** recorded in DMSO-*d*6.

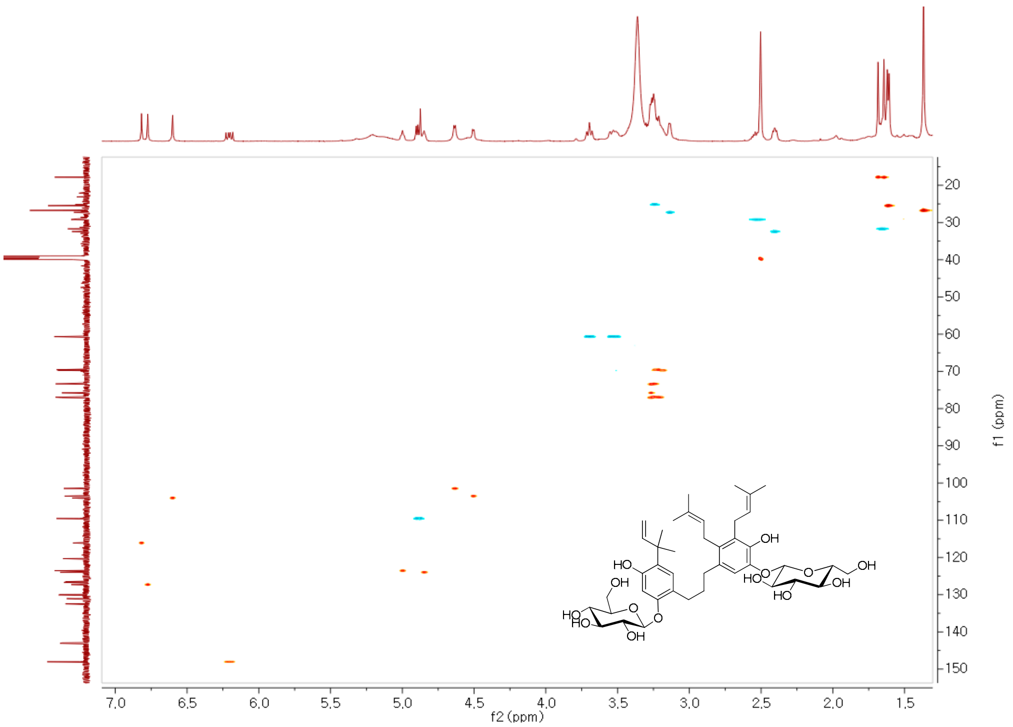

Figure S27. HSQC spectrum of compound **7** recorded in DMSO-d_6.

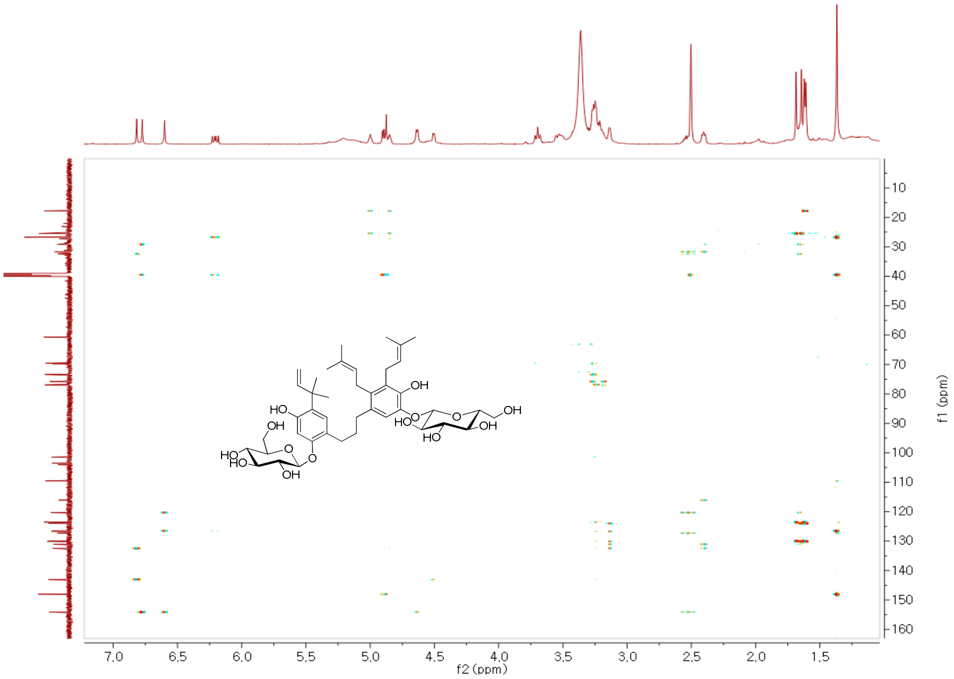

Figure S28. HMBC spectrum of compound **7** recorded in DMSO-d_6.

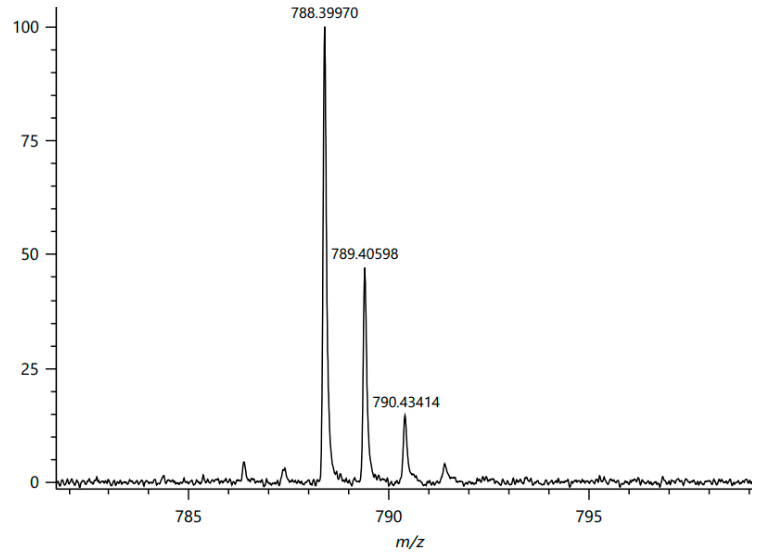

Figure S29. The HRFDMS spectrum of compound **7**.

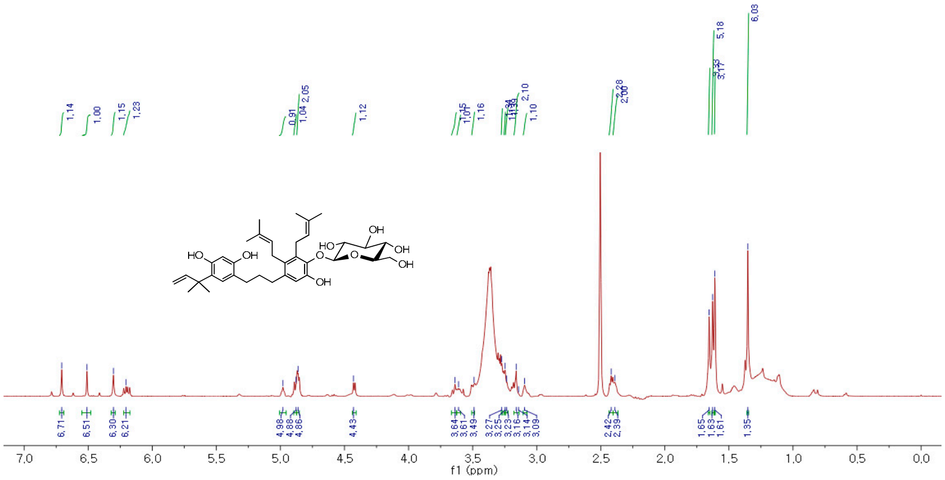

Figure S30. ^{1}H NMR spectrum of compound **8** recorded at 600 MHz in DMSO-d_6.

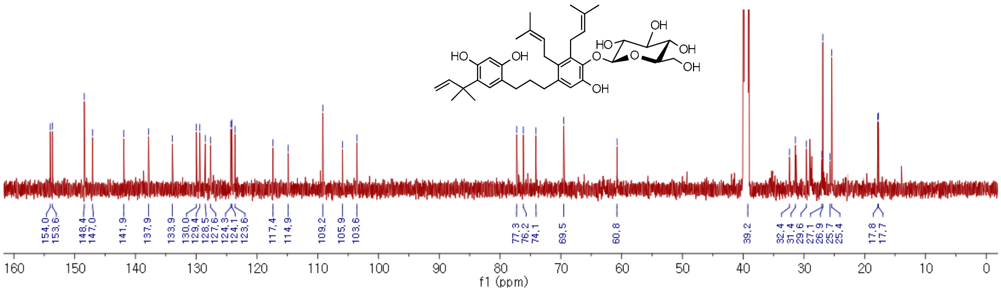

Figure S31. ^{13}C NMR spectrum of compound **8** recorded at 150 MHz in DMSO-d_6.

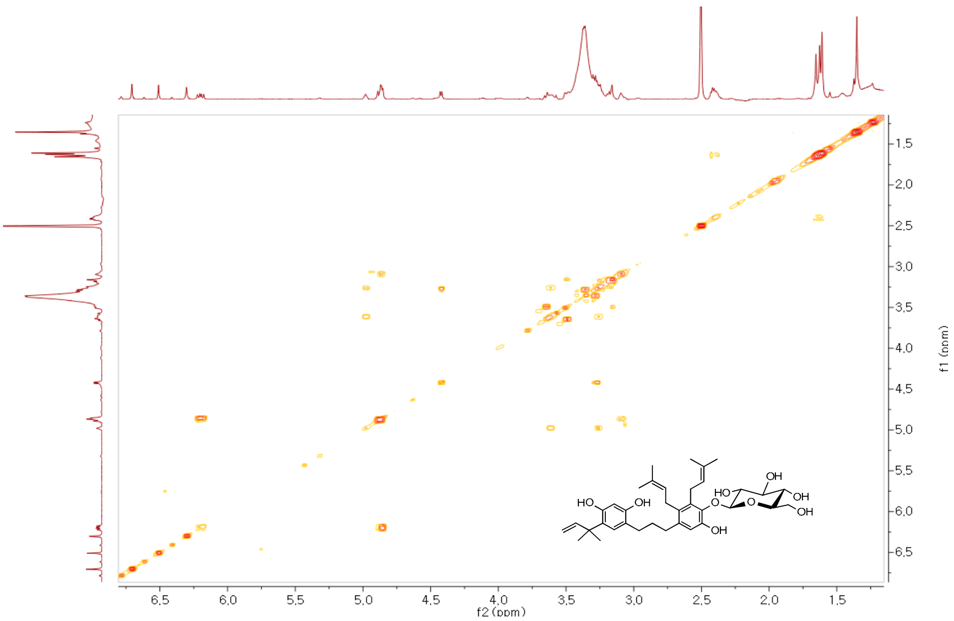

Figure S32. COSY spectrum of compound **8** recorded in DMSO-*d*6.

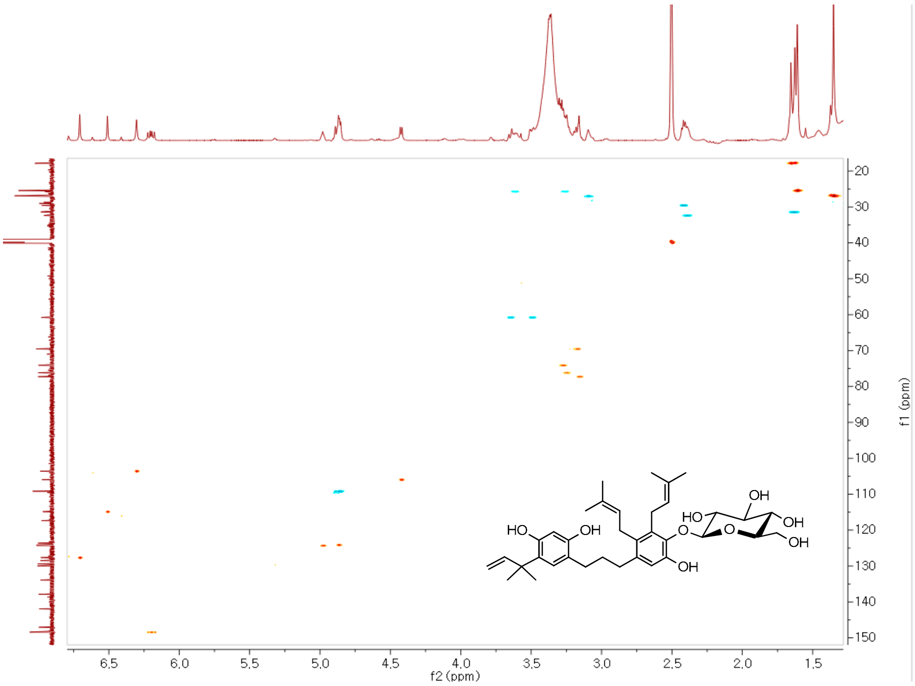

Figure S33. HSQC spectrum of compound **8** recorded in DMSO-*d*6.

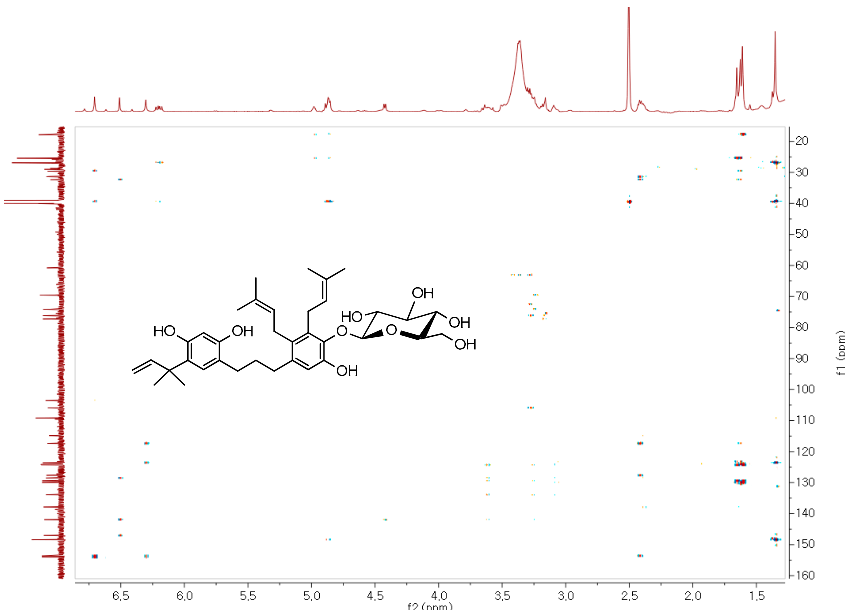

Figure S34. HMBC spectrum of compound **8** recorded in DMSO-d_6.

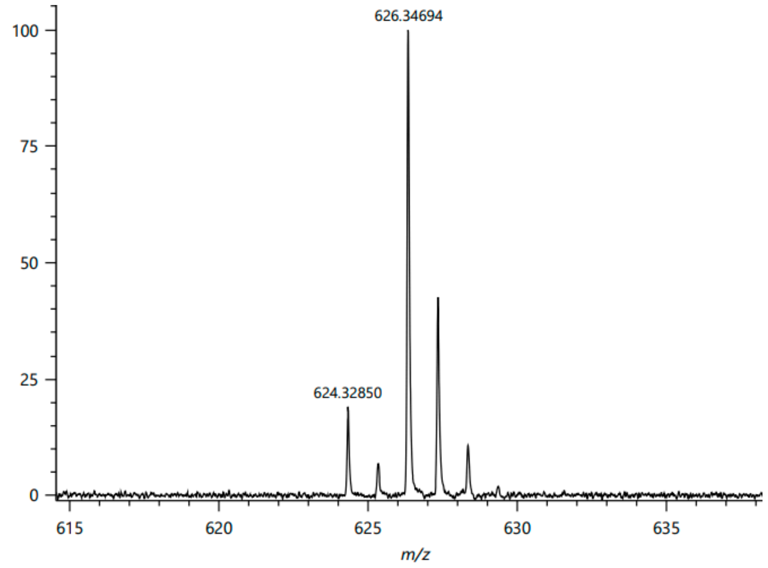

Figure S35. The HRFDMS spectrum of compound **8**.

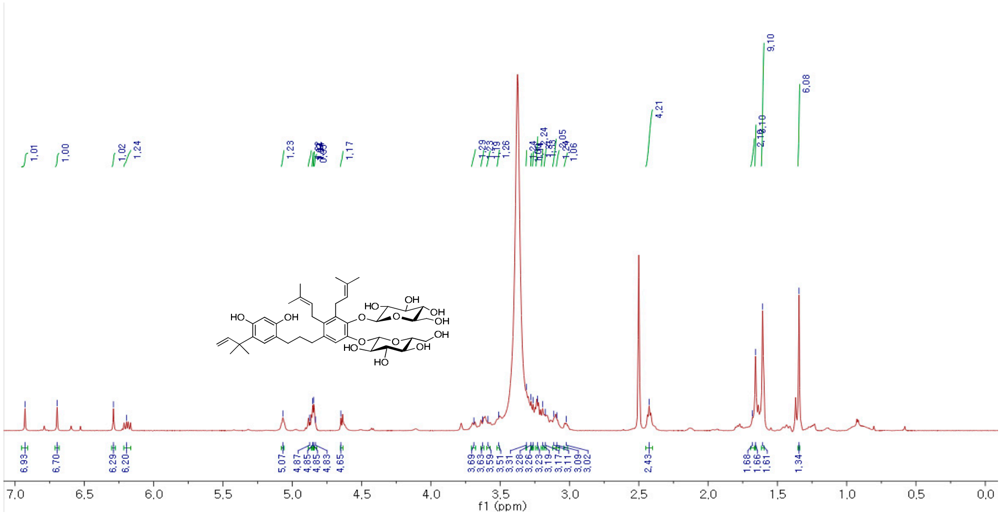

Figure S36. ^{1}H NMR spectrum of compound **9** recorded at 600 MHz in DMSO-d_6.

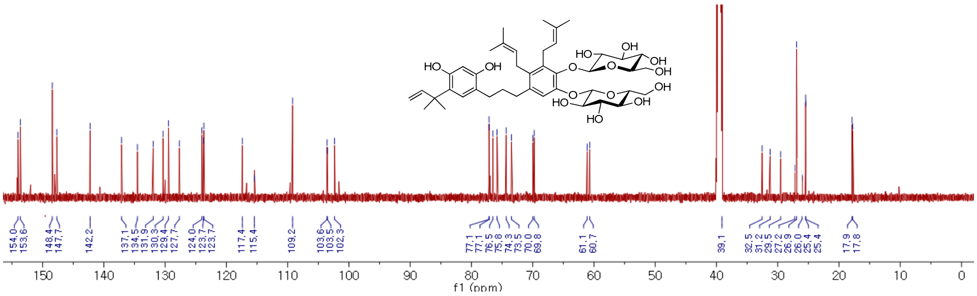

Figure S37. ^{13}C NMR spectrum of compound **9** recorded at 150 MHz in DMSO-d_6.

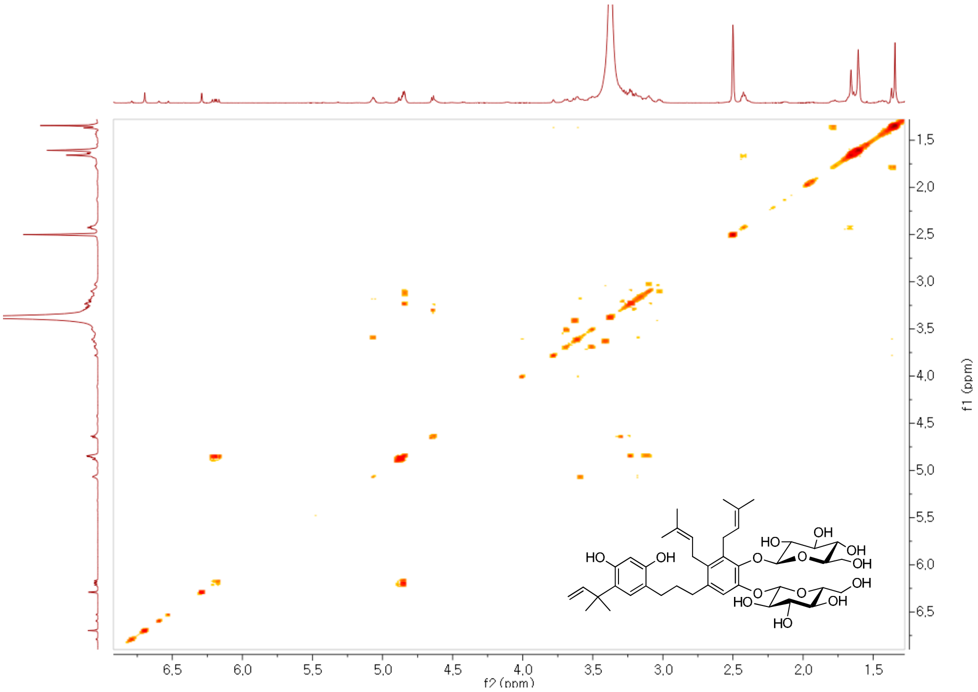

Figure S38. COSY spectrum of compound **9** recorded in DMSO-d_6.

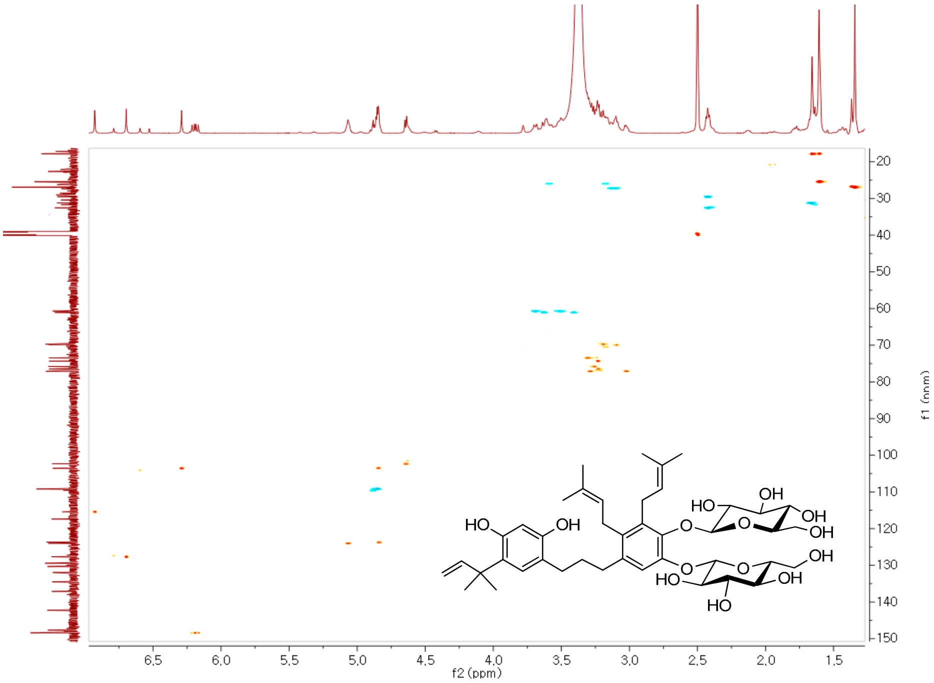

Figure S39. HSQC spectrum of compound **9** recorded in DMSO-d_6.

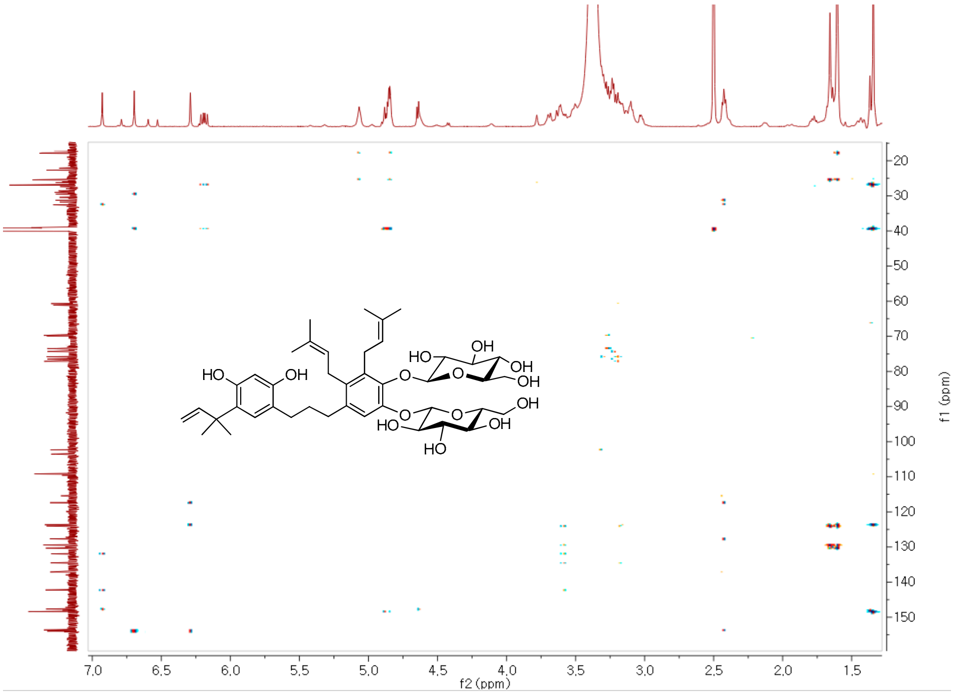

Figure S40. HMBC spectrum of compound **9** recorded in DMSO-d_6.

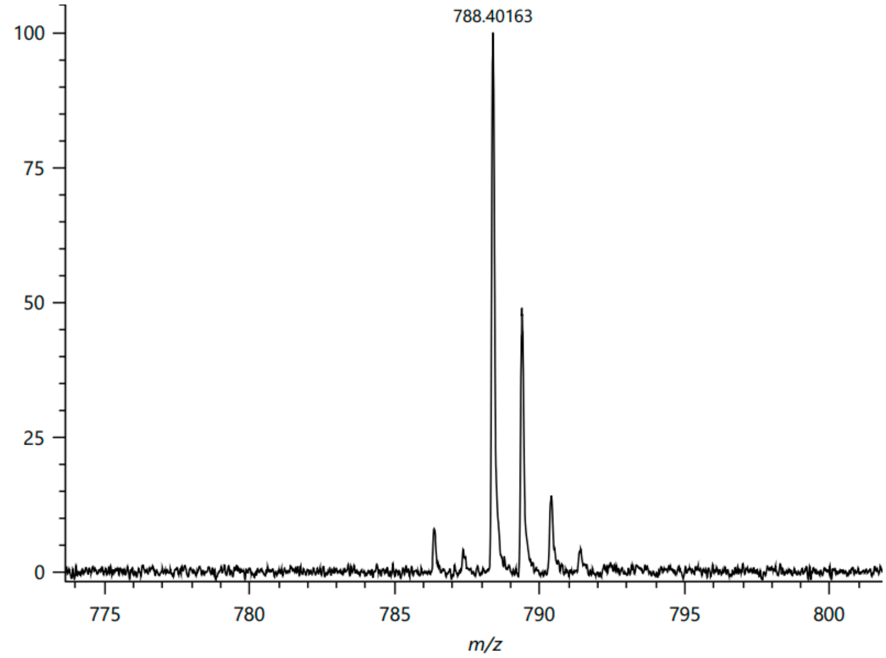

Figure S41. The HRFDMS spectrum of compound **9**.

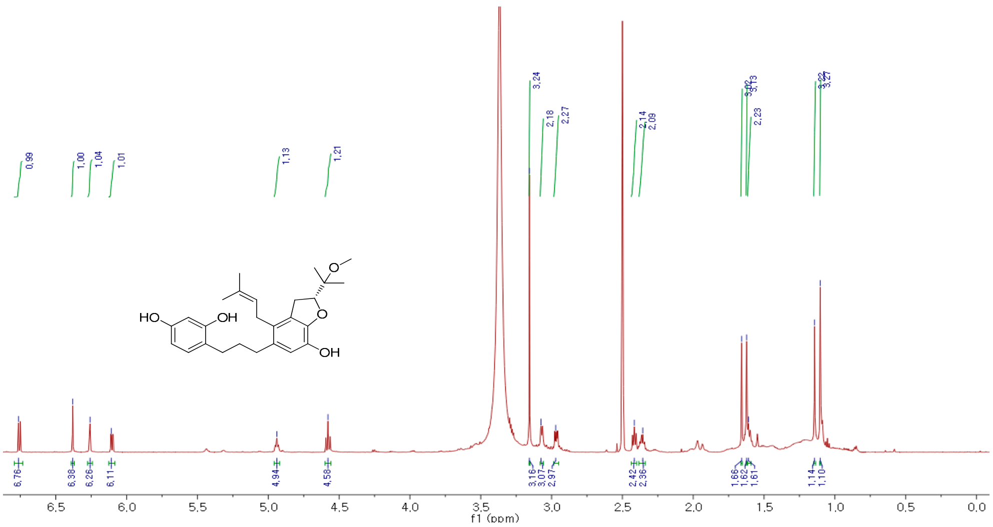

Figure S42. ^{1}H NMR spectrum of compound **10** recorded at 600 MHz in DMSO-*d*6.

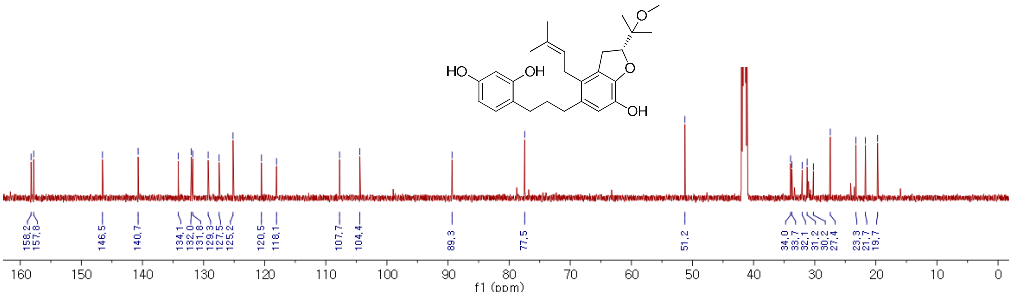

Figure S43. ^{13}C NMR spectrum of compound **10** recorded at 150 MHz in DMSO-*d*6.

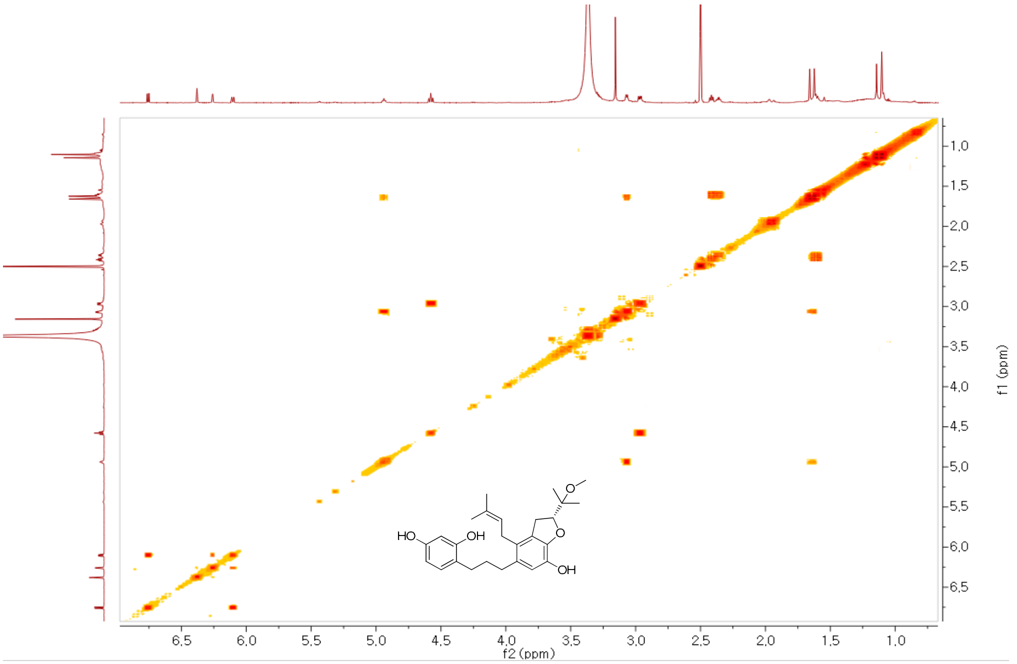

Figure S44. COSY spectrum of compound **10** recorded in DMSO-*d*6.

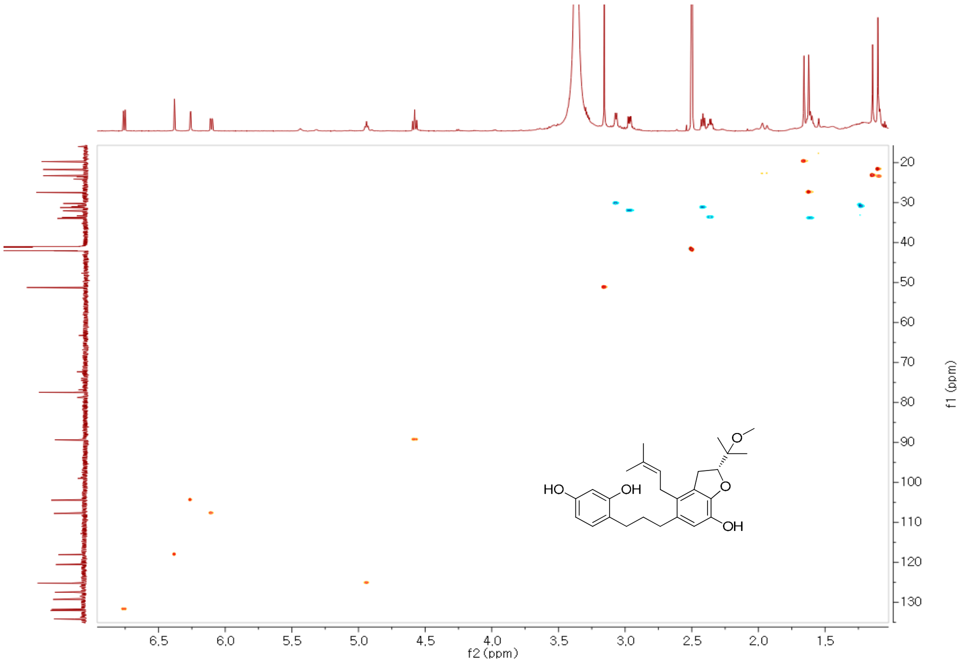

Figure S45. HSQC spectrum of compound **10** recorded in DMSO-d_6.

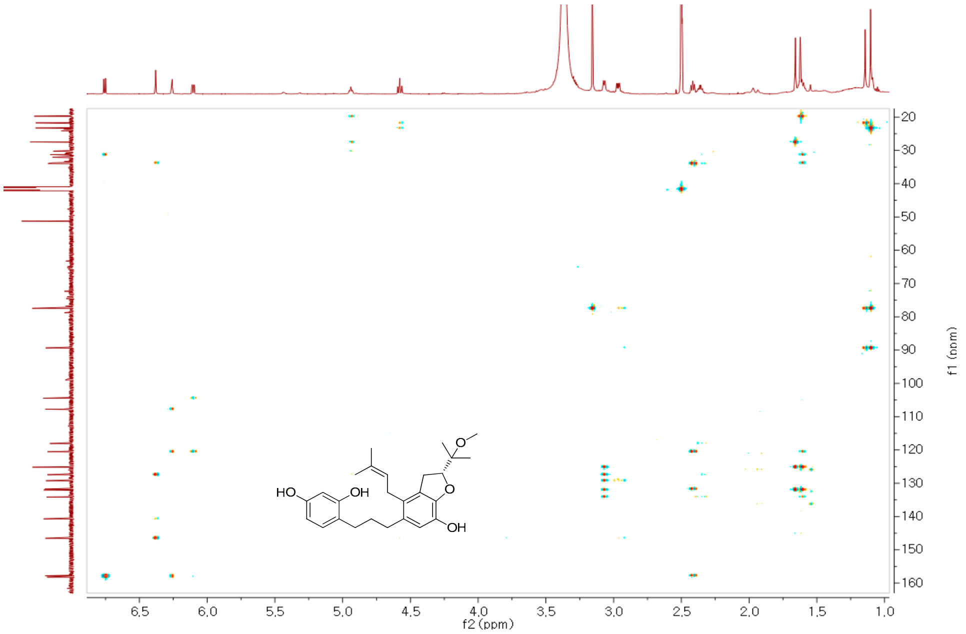

Figure S46. HMBC spectrum of compound **10** recorded in DMSO-d_6.

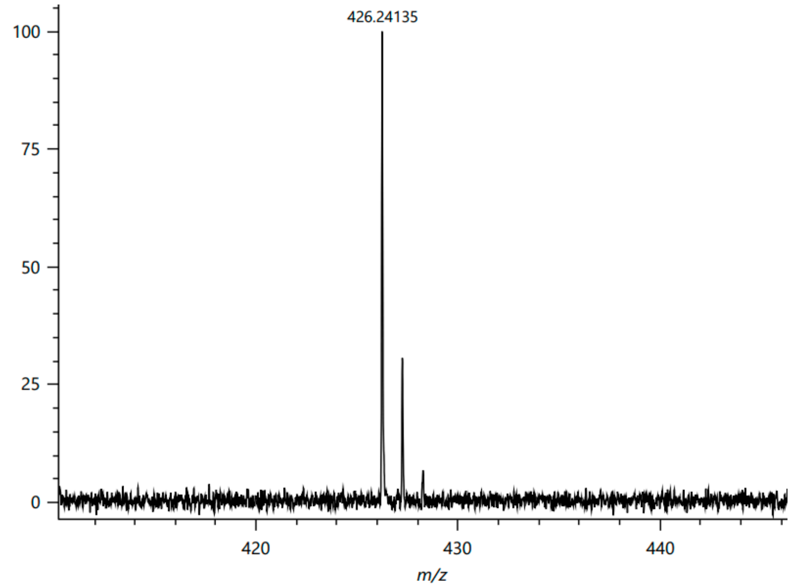

Figure S47. The HRFDMS spectrum of compound **10**.

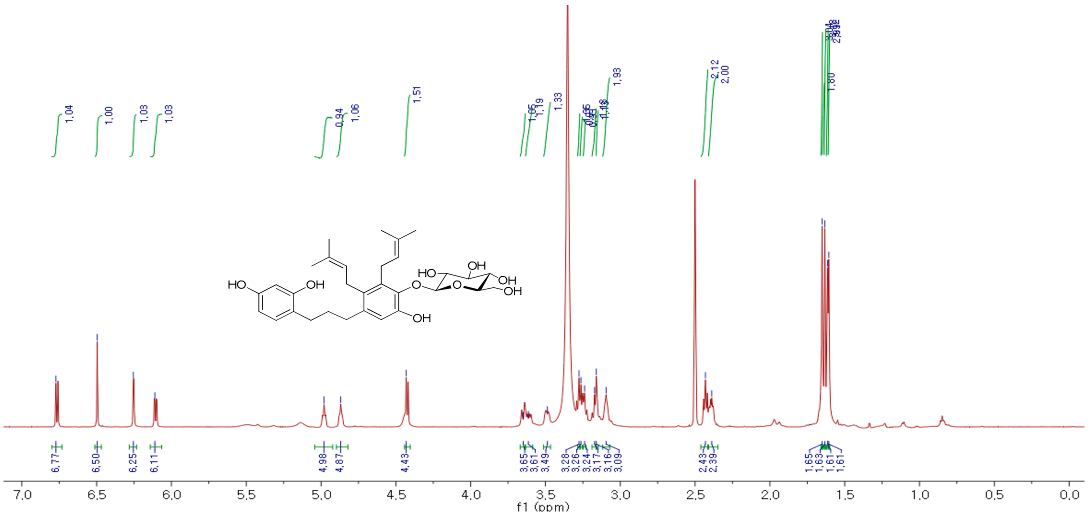

Figure S48. ^{1}H NMR spectrum of compound **11** recorded at 600 MHz in DMSO-d_6.

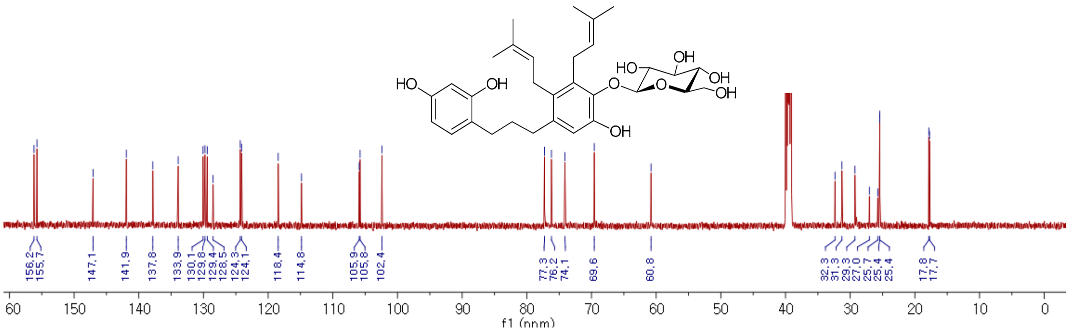

Figure S49. ^{13}C NMR spectrum of compound **11** recorded at 150 MHz in DMSO-d_6.

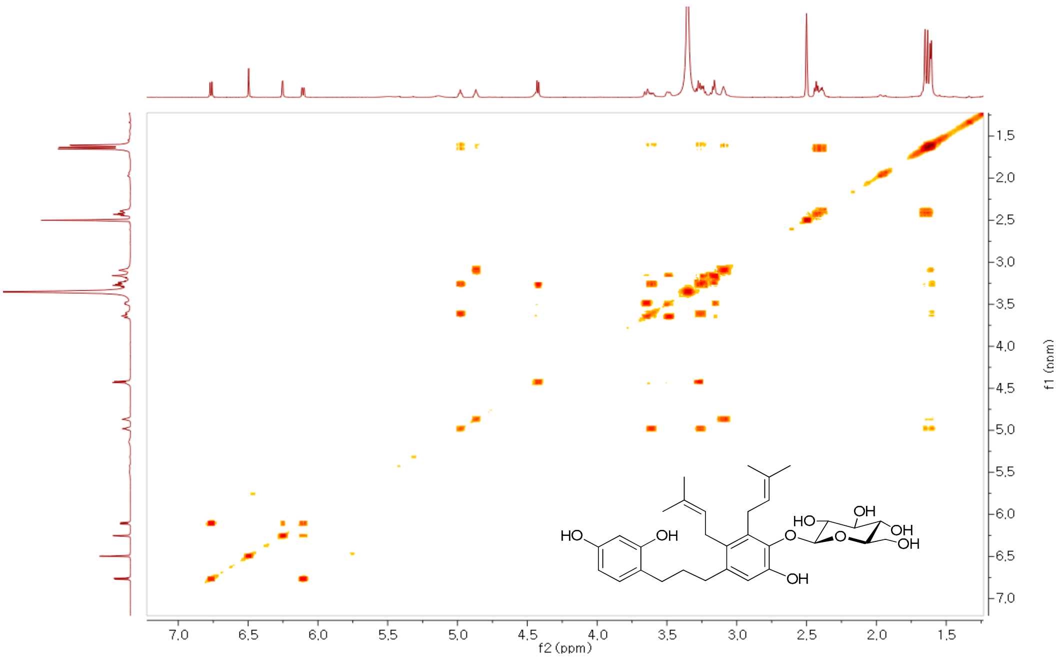

Figure S50. COSY spectrum of compound **11** recorded in DMSO-d_6.

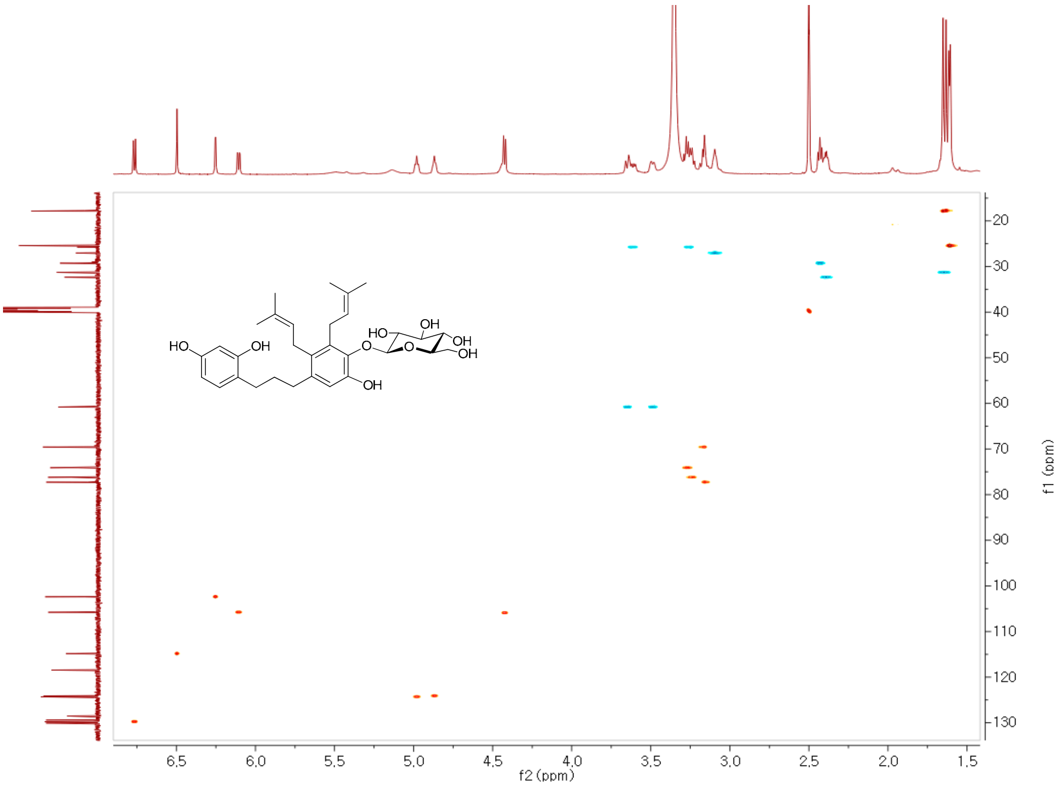

Figure S51. HSQC spectrum of compound **11** recorded in DMSO-d_6.

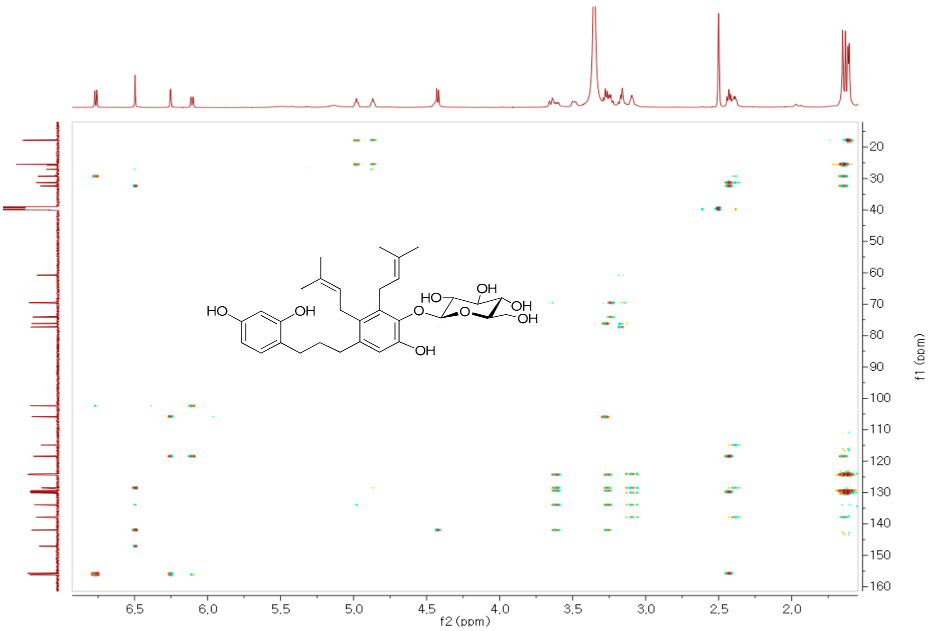

Figure S52. HMBC spectrum of compound **11** recorded in DMSO-*d*6.

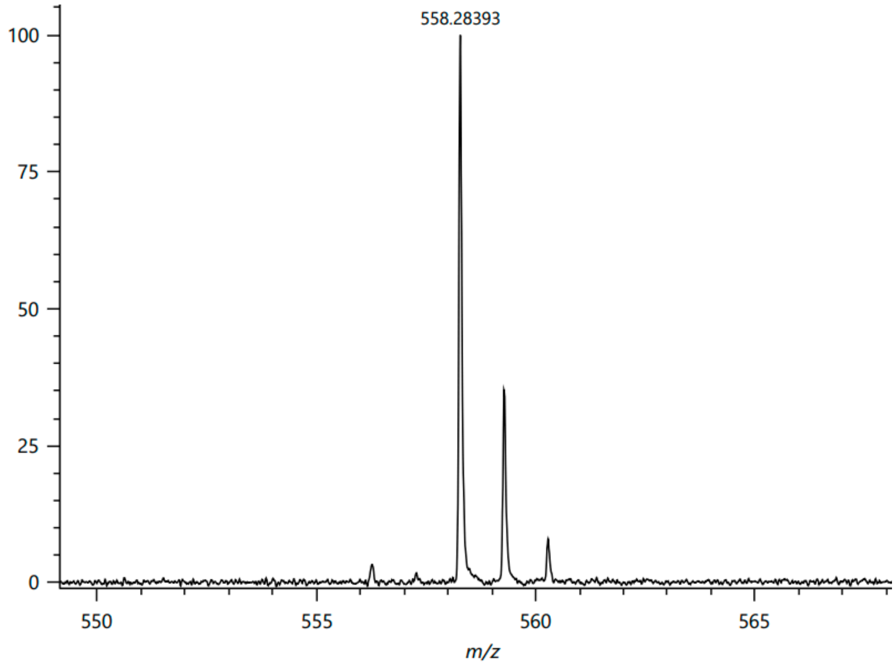

Figure S53. The HRFDMS spectrum of compound **11**.
